# FANCD2 maintains replication fork stability during misincorporation of the DNA demethylation products 5-hydroxymethyl-2’-deoxycytidine and 5-hydroxymethyl-2’-deoxyuridine

**DOI:** 10.1038/s41419-022-04952-0

**Published:** 2022-05-27

**Authors:** María José Peña-Gómez, Paula Moreno-Gordillo, Milda Narmontė, Clara B. García-Calderón, Audronė Rukšėnaitė, Saulius Klimašauskas, Iván V. Rosado

**Affiliations:** 1 Centro Andaluz de Biología Molecular y Medicina Regenerativa (CABIMER) Universidad de Sevilla-CSIC-Universidad Pablo de Olavide, Seville, 41092 Spain; 2 Institute of Biotechnology, Life Sciences Center, Vilnius University, Vilnius, 10257 Lithuania; 3 Instituto de Biomedicina de Sevilla (IBiS)/CSIC/Universidad de Sevilla/Campus Hospital Universitario Virgen del Rocío, Seville, 41013 Spain; 4 Departamento de Genética, Universidad de Sevilla, Seville, 41012 Spain

**Keywords:** DNA damage and repair, DNA replication

## Abstract

Fanconi anemia (FA) is a rare hereditary disorder caused by mutations in any one of the FANC genes. FA cells are mainly characterized by extreme hypersensitivity to interstrand crosslink (ICL) agents. Additionally, the FA proteins play a crucial role in concert with homologous recombination (HR) factors to protect stalled replication forks. Here, we report that the 5-methyl-2’-deoxycytidine (5mdC) demethylation (pathway) intermediate 5-hydroxymethyl-2’-deoxycytidine (5hmdC) and its deamination product 5-hydroxymethyl-2’-deoxyuridine (5hmdU) elicit a DNA damage response, chromosome aberrations, replication fork impairment and cell viability loss in the absence of FANCD2. Interestingly, replication fork instability by 5hmdC or 5hmdU was associated to the presence of Poly(ADP-ribose) polymerase 1 (PARP1) on chromatin, being both phenotypes exacerbated by olaparib treatment. Remarkably, *Parp1*^−/−^ cells did not show any replication fork defects or sensitivity to 5hmdC or 5hmdU, suggesting that retained PARP1 at base excision repair (BER) intermediates accounts for the observed replication fork defects upon 5hmdC or 5hmdU incorporation in the absence of FANCD2. We therefore conclude that 5hmdC is deaminated in vivo to 5hmdU, whose fixation by PARP1 during BER, hinders replication fork progression and contributes to genomic instability in FA cells.

## Introduction

FA is a rare genetic disease featured by hematological, developmental and cellular defects that threaten human life [1, 2]. The defects underlying the FA syndrome can be ascribed to a molecular pathway that maintains and protects the genome integrity [3, 4] and ensures the faithful transmission of genetic information from a mother to a daughter cell [5]. The FA/BRCA pathway is considered a S-phase specific homologous recombination pathway which repairs ICLs induced by agents (e.g., cisplatin, mitomycin C, aldehydes) that join together the two strands of DNA [6–8]. Replication forks frequently stall at sites difficult to replicate, leading to aberrant structures which accumulate single-stranded DNA (ssDNA) and elicit replication stress responses [9]. The FA pathway also maintains replication fork stability upon replication stress to ensure cell survival [4, 8], and shares several protective functions with homologous recombination (HR) factors against replication failure, including origin firing and replication fork restart, stabilization and protection of stalled forks against nucleolytic degradation. Under these conditions, the ATR kinase activated by ssDNA present at stalled replication forks [10] phosphorylates the checkpoint kinase CHK1, which prevents origin firing and facilitates RAD51-mediated homologous recombination (HR) [11]. Stalled replication forks then undergo reversible remodeling to avoid replisome collapse, which at the end relies on the homologous recombination (HR) repair factors such as FANCD2, RAD51, BRCA1 and BRCA2 to protect nascent DNA from nucleolytic degradation mediated by MRE11 or DNA2 [12–15].

Compelling evidence demonstrate that chemical modification of DNA bases can occur enzymatically or spontaneously in an uncontrolled manner. As such, formaldehyde forms base adducts and crosslinked DNA bases amongst other lesions [16], which stem at the basis of hematopoietic stem cell loss, bone marrow failure and cancer predisposition in FA mice and patients [17–21], although the specific formaldehyde-derived lesion specifically repaired by the FA pathway still remains elusive. Furthermore, formaldehyde reaction with free DNA bases causes N-hydroxymethylated monoadducts [22] that can potentially contaminate the free nucleotide pool, leading to the formation of crosslinks between adjacent purines [22] and altering the epigenetic information throughout the formation of 5hmdC mediated by DNMTs [23, 24]. 5hmdC arises in genomic DNA via TET-mediated oxidation of 5mdC during cytosine demethylation and regulates gene transcription, differentiation and cell fate decisions. 5hmdC is further converted into 5-formylcytosine (5fdC) and 5-carboxycytosine (5cadC) by a TET-mediated stepwise processive oxidation, these latter being actively removed by the BER pathway involving Thymine DNA glycosylase (TDG) [25, 26], which finally replace them by an unmodified cytosine. 5hmdC marks replication origin and DNA repair sites [27, 28] and it is deaminated to 5hmdU at the nucleotide monophosphate step or once in genomic DNA by the cytidine deaminases DCTD and CDA, or AID respectively, which readily misincorporates by replicative polymerases perturbing Watson and Crick base pairing [29]. In fact, 5hmdU is actively removed by SMUG1 and TDG glycosylases [30, 31]. However, whether these 5mdC demethylation DNA bases 5hmdC or 5hmdU threats replication integrity during processing and removal by the base excision machinery is not completely understood.

Herein, we describe a protective role of the FA pathway against 5hmdC and 5hmdU genotoxicity. Our findings reveal that FANCD2 protects replication fork integrity from 5hmdC-induced chromosome instability. 5hmdC supplementation led to a 2000-fold increase of its deamination analog 5hmdU in the genome, and 5hmdU exposure recapitulated the phenotypes caused by 5hmdC, suggesting that 5hmdC-derived 5hmdU accumulates in the genome and perturbs replication dynamics in the absence of FANCD2. Mechanistically, 5hmdU or 5hmdC-mediated fork impairment and genomic instability phenotypes were exacerbated by trapped PARP1, suggesting that PARP1 retained or trapped on chromatin during the processing and removal of incorporated 5hmdU challenges replication fork integrity, which necessitates FANCD2 to maintain its stability.

## Methods

### Cell lines and reagents

The *Fancd2*^+/−^ C57/BL6 sv129 hybrid background mice were timed mated, and murine embryonic fibroblasts (MEFs) isolated from pups at E13.5, confirmed by PCR [32] and transformed with a plasmid containing the SV40 large T antigen. *Wild type*, *Fancd2*^−/−^, *Parp1*^+/+^ or *Parp1*^−/−^ MEFs and human eHAP were grown in DMEM and IMDM medium supplemented with 10% FBS, Penicillin/streptamycin (Gibco) respectively. Patient-derived HSC72 (FANCA-deficient) and NV012 human cell lines were grown in RPMI1640 medium supplemented with 10% FBS, Penicillin/streptomycin (Gibco) and 50 μM 2-mercartoethanol (Merck). 5dC (D3897; Sigma), 5mdC (PY 7635; B&A), 5hmdC (PY 7588; B&A), 5fdC (PY 7589; B&A), 5cadC (PY 7593; B&A), BrdU (B5002; Sigma), CldU (Sigma, C6891), IdU (Sigma, I7125), EdU (Thermo Scientific, A10044) were dissolved in PBS (10 mM stock solution), and olaparib (Selleckchem, S1060) was dissolved in DMSO (10 mM stock solution), and added to cell cultures as indicated. The antibodies used were against ser139-H2AX (Millipore, 05-636), PAR (Millipore, MABE1016), FANCD2 (Novus, NB100-316), ser345-CHK1 (Cell Signaling 2348), CHK1 (sc-8408), ERCC1 (sc-8408), PCNA (sc-56), Lamin A/C (sc-376248), BrdU (AbSerotec, OBT0030), BrdU (BD Bioscience, 347580), α-tubulin (Sigma, T9026), ser4/ser8 RPA32 (Bethyl, A300-245A).

### Immunofluorescence microscopy

For immunofluorescence analysis, 5 × 10^4^ cells were seeded in coverslips and incubated with the chemicals for the indicated times. Cells were permeabilized using 0.25% Triton X-100 in PBS at 4 °C for 2 min and fixed with 4% formaldehyde in PBS at 4 °C for 15 min. Then, cells were blocked in PBS + 0.3% Tween20 + 3%BSA and incubated with the primary antibody of interest overnight at 4 °C. Next day, cells were incubated with alexa fluor-labeled secondary antibody and 4´6-diamidine-2-phenylindole dihydrochloride (DAPI) was added at 0.1 mg/mL for 1 h at RT in dark conditions. Finally, coverslips were mounted in ProLong® Gold Antifade Reagent. For EdU immunofluorescence, cells were incubated with 10 µM EdU for 2 h. Cells were permeabilized, fixed and blocked as previously described, and click-it reaction (100 mM Tris-HCl 1.5 M pH 7.5, 1 µM 488 fluorescence azide, 1 mM CuSO_4_, 100 mM ascorbic acid) was carried out for 30 min at RT in dark conditions.

For immunostaining quantitation purposes of images containing DAPI, γ-H2AX and PAR staining was used as a template to make an automated mask around nuclei, applied to the γ-H2AX or PAR channels, and the signal intensity median value was calculated and foci number was calculated using *2a.Red channel foci Analysis* macro. Background noise signal was averaged and subtracted from the nuclear signal. All immunostained images were quantitated using ImageJ 6.0 software and plotted using GraphPad Prism 6.0 software.

### Analysis of genomic 5hmdC and 5hmdU by HPLC-MS/MS

HPLC-MS/MS analysis was performed as described by Tomkuviene et al. [33] with modifications. DNA (2–4 µg per sample) hydrolyzates were analyzed on an integrated HPLC/ESI-MS/MS system (Agilent 1290 Infinity/ 6410 Triple Quad) equipped with a Supelco DiscoveryHS C18 column (75 × 2.1 mm, 3 μm) by elution with a linear gradient of solvents A (0.0075% formic acid in water) and B (0.0075% formic acid in acetonitrile) at a flow of 0.3 ml/min at 30 °C as follows: 0–6 min, 0% B; 6–18 min, 10% B; 18–20 min, 100% B. Mass spectrometer was operating in the positive ion mode at a capillary voltage of 1800 V; drying gas temperature 300 °C and flow rate 10 l/min. Data was analyzed using Agilent MassHunter software.

### Isolation of Proteins on Nascent DNA (iPOND)

iPOND was performed as described by Sirbu and colleagues [34], with few modifications to improve protein capture. MEFs were labeled with 10 μM EdU (Life Technologies) for 20 min and treated with 5hmdC (160 μM) or HU (1 mM) for 3 h. Cells were permeabilized and subjected to click-iT reaction using Biotin azide (Invitrogen). Cells were lysed and sonicated at 4 °C. Finally, by incubation with streptavidin-magnetic sepharose beads (Abcam) overnight at 4 °C. Bound proteins from the EdU-labeled pulled down DNA were eluted by boiling in SDS sample buffer (2xSB).

### DNA fiber technique

For replication fork blockage purpose in the presence of 5hmdC, 5hmdU or their combinations with olaparib, we modified the incubation periods to 60 min to maximize 5hmdC or 5hmdU uptake by cells. Cells were firstly pulsed with 25 μM CldU for 30 min, washed and incubated in medium containing 50 μM IdU in the presence of 5hmdC, or 5hmdU for 60 min. DNA fibers were incubated with rat anti-BrdU antibody (AbD Serotec, 1:1000) and mouse anti-BrdU antibody (Bu20a,1:500) and stained with goat anti-rat alexa-488 (Invitrogen Inc.) and goat anti-mouse Alexa555 antibody (Invitrogen Inc.) for 2 h respectively and mounted using Prolong Antifade medium (Invitrogen Inc.).

### CRISPR/Cas9-mediated gene disruptions in eHAP and MEFs cell lines

Guide sequences targeting exon 2 of the human *FANCD2* gene were obtained using the CRISPRsearch tool from the Sanger Institute. FD2-4 5-TACTGGAGGCATCTTCTGTCAGG-3´; FD2-5 5´- AATGACTAGATACTTACTGGAGG-3´. DNA oligos were cloned into pSpCas9(BB)-2A-GFP (PX458, a gift from Dr. Feng Zhang, Addgene plasmid # 48138) and confirmed by sanger sequencing. After transfection, single-cell sorted GFP positive cells were plated and individual clones analyzed for FANCD2 deletions and confirmed by DNA sequencing and western blotting.

### Cell cycle analyses

For acute cell cycle experiments, 1.5 × 10^5^ cells were seeded the day before, exposed to 5dC, 5mdC or 5hmdC (100 μM) for 30 min, washed, incubated in fresh media and analyzed at 48 h. For prolonged exposure, 5 × 10^5^ cells were exposed to 5dC, 5mdC or 5hmdC (10 μM) for 1, 4, 8, 12 or 16 h. Cells were fix and analyzed by flow citometry.

### Statistical analysis

The number of independent technical repeats (*n*) are indicated in figure legends. Data are shown as mean ± standard deviation (s.d.) or median. The nonparametric Mann–Whitney test or Student’s *t* test were employed to determine statistical significance (*p < 0.05,**p < 0.01,***p < 0.001,****p < 0.0001). Data analysis were performed in GraphPad Prism 9.

## Results

### 5hmdC elicits DNA damage response and blocks replication fork progression in *FancD2*^*−/−*^ cells

**Fig. 1:**
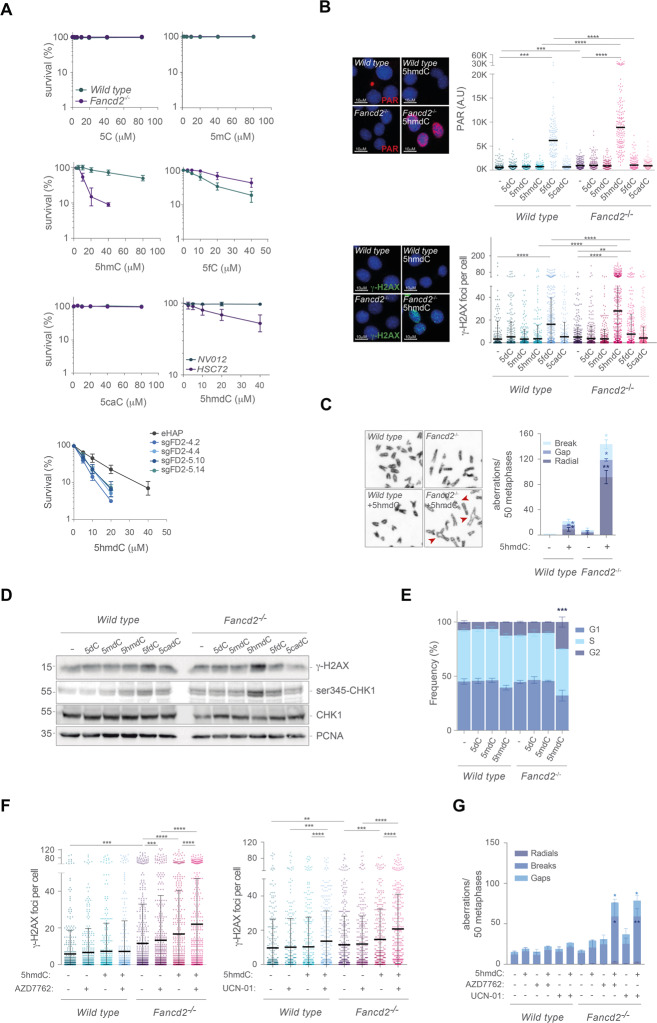
5hmdC exposure induces genome instability in *Fancd2*^*−/−*^ cells. **A** Cell proliferation assay of *wild type*, *Fancd2*^−/−^ exposed to the indicated dose of 5dC, 5mdC, 5hmdC, 5fdC and 5cadC for 3 days (*n* = 4, mean ± s.d.). Cell proliferation assay of a lymphoblast FANCA-deficient patient-derived *HSC72*, *NV012* cell lines and *FANCD2*^−^ KO eHAP CRISPR clones exposed to the indicated doses of 5hmdC for 3 days (*n* = 4, mean ± s.d.). **B** *Top left*, representative PAR (red) immunofluorescence images of *wild type* and *Fancd2*^−/−^ cells exposed to 5hmdC (10 μM) for 16 h. DAPI (blue) stains nuclear DNA. *Top right*, plot depicting PAR mean intensity signal per nucleus (*n* = 3, Mann–Whitney test; central line represents median value). *Bottom left*, representative γ-H2AX (green) immunofluorescence images of *wild type* and *Fancd2*^−/−^ cells exposed to 5hmdC (10 μM) for 16 h. DAPI (blue) stains nuclear DNA. Bottom *right*, plot depicting γ-H2AX foci per nucleus (*n* = 3, Student’s *t* test; central line represents mean ± s.d.). **C** *Left*, representative images of chromosome aberration test (red arrowhead) from *wild type* and *Fancd2*^−/−^ cells following 5hmdC treatment (10 μM) for 40 h. *Right*, bar plot of breakdown of the different types of chromosomal aberrations (*n* = 150 of each of 3 biological replicates, Student’s *t* test; bar represents mean ± s.e.m.). **D** Western blot of *wild type* and *Fancd2*^−/−^ MEFs extracts to detect γ-H2AX, ser345-CHK1, total CHK1, and PCNA (loading control) after exposure to 5dC, 5mdC, 5hmdC, 5fdC or 5cadC (10 μM) for 16 h. **E** Bar plot depicting frequency of G1, S or G2 populations of *wild type* and *Fancd2*^−/−^ cells exposed to 5dC, 5mdC and 5hmdC (100 μM) for 30 min and subsequently analyzed after 48 h in culture (*n* = 3, Student’s *t* test; bar represents mean ± s.d.). **F** *Left*, plot depicting γ-H2AX foci per nucleus of *wild type* and *Fancd2*^−/−^ cells exposed to AZD7762 (2.5 nM), 5hmdC (10 μM) or combination of both for 16 h (*n* = 3, Mann–Whitney test; central line represents mean value). *Right*, as in *Left* but AZD7762 was substituted by UCN-01 (2.5 nM). **G** Bar plot of breakdown of the different types of chromosomal aberrations from *wild type* and *Fancd2*^−/−^ cells treated with 5hmdC (10 μM), AZD7762 (2.5 nM), UCN-01 (2.5 nM) or combinations for 48 h. AZD7762 or UCN-01 were added 24 h before harvesting the cells (*n* = 100 of each of 2 biological replicates, Student’s *t* test; bar represents mean ± s.d).

Formaldehyde is clearly genotoxic to FA cells [17, 18, 35], and reacts with DNA bases in vitro and in vivo forming N-hydroxymethylated bases [36], including 5hmdC [23]. Therefore, we sought to investigate the sensitivity of *Fancd2*^−/−^ cells to 5hmdC and other cytosine analogs naturally present in genomic DNA such as 5mdC, 5fdC and 5cadC. Despite of being previously described as innocuous [37], 5hmdC but not 5dC, 5mdC, 5fdC or 5cadC caused a marked sensitivity to *Fancd2*^−/−^ murine cells (Fig. 1A). This sensitivity was corroborated in a FANCA patient-derived lymphoblastoid cell line (HSC72), in a FANCD2^−^ CRISPR-Cas9 knocked out eHAP cell line (Fig. 1A and Supplementary Fig. 1) and in *FANCC*^−^, *FANCF*^−/−^*, FANCL*^−/−^ or *FANCG*^−/−^ DT40 cells (Supplementary Fig. 2A), suggesting that loss of a functional FA/BRCA pathway sensitizes cells to 5hmdC, a feature conserved across different vertebrate species. Unlike other cytosine analogs, 5hmdC exposure led to heightened levels of nuclear PARylation, probably due to increased PARP activity during repair of 5hmdC-mediated DNA damage by BER (Fig. 1B), leading to extensive γ-H2AX foci formation and suggesting that 5hmdC increased double strand break (DSBs) formation. Consistently, 5hmdC exposure increased the frequency of chromosomal aberrations in the absence of FANCD2 (Fig. 1C), that presumably arose from misrepaired 5hmdC DNA lesions thorough BER intermediates during replication. To address the contribution of 5hmdC-mediated DNA damage to cell cycle checkpoint activation, we examined cell cycle profile of cells upon 5hmdC exposure. Despite the fact that 5dC, 5mdC or 5hmdC exposure (10 μM upon 16 h) hardly affected cell cycle profiles (Supplementary Fig. 3A), a time course experiment showed a mild perturbation of the S phase by 5dC, 5mdC and 5hmdC, which were alleviated at later time points (8 and 12 h) (Supplementary Fig. 3B). However, exposure to 5hmdC induced ser345-CHK1 phosphorylation in *Fancd2*^*−/−*^ cells concomitantly to γ-H2AX, suggesting that DSBs arose during replication stress at S phase (Fig. 1D). Therefore, we challenged cells with high dose 5hmdC (100 μM for 30 min) and analyzed cell cycle after 48 h. Strikingly, exposure to 5hmdC, but not to 5dC or 5mdC, led to a G2/M cell cycle arrest of *Fancd2*^−/−^ cells (Fig. 1E), consistent with a checkpoint activation by unrepaired DNA damage. In fact, the CHK1 inhibitors AZD7762 or UCN-01 overcame G2/M cell cycle arrest induced by 5hmdC (Supplementary Fig. 3C) at expenses of increasing γ-H2AX foci formation (Fig. 1F), resulting in a further increase frequency of 5hmdC-mediated chromosomal abnormalities (Fig. 1G). In addition, 5hmdC exposure promoted FANCD2 monoubiquitylation in human eHAP and chicken DT40 cell lines in a dose-dependent manner (Supplementary Fig. 2B), indicating that 5hmdC was taken by cells, activated FANCD2 monoubiquitylation and induced chromosomal instability and lethality in FA cell lines, and suggest that the cytosine demethylation nucleoside 5hmdC elicits a classical DNA damage response in FA cells, unveiling a novel role of the FA pathway in maintaining genome integrity and survival.

**Fig. 2:**
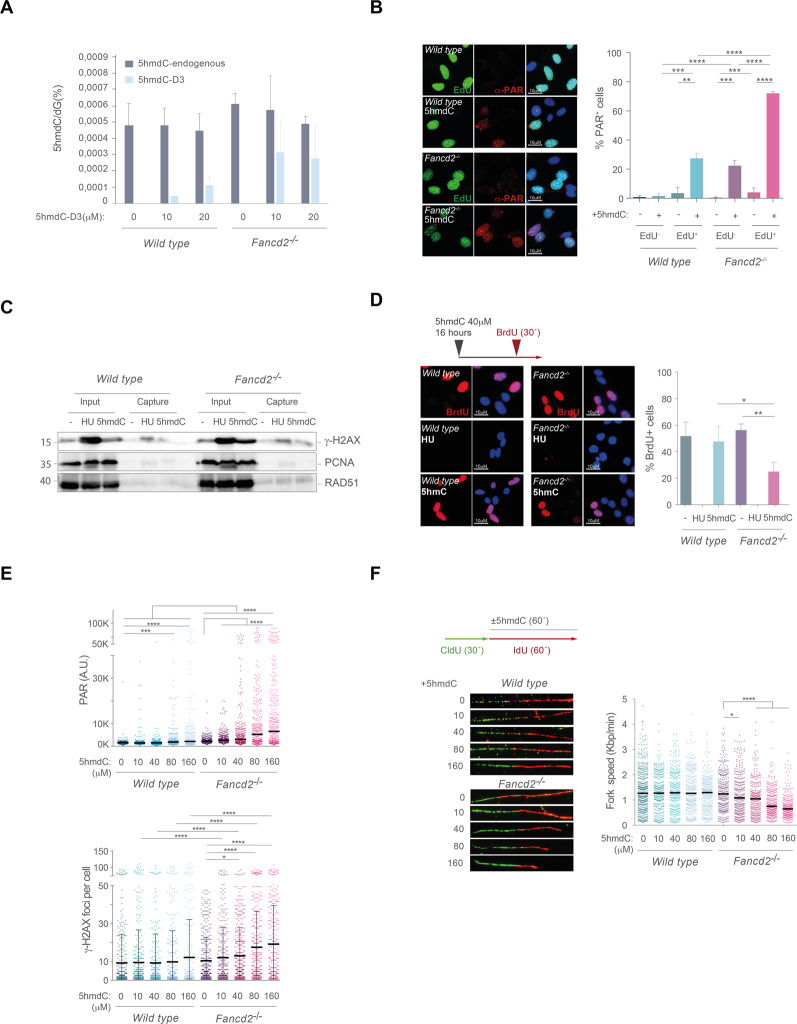
5hmdC-induced DNA damage associates to S-phase and impairs replication fork progression. **A** HPLC-MS/MS quantitation of endogenous and exogenous 5hmdC levels in genomic DNA samples of *wild type* and *Fancd2*^−/−^ cells exposed to D3-labeled 5hmdC (0, 10 and 20 μM) for 16 h. *Wild type* cells showed a 0.49 vs 4.8 exogenous vs endogenous 5hmdC per 10^4^ dG upon 10 μM 5hmdC-D3 exposure; 1.2 5hmdC-D3 vs 4.5 5hmdC per 10^4^ dG for 20 μM exposure). *Fancd2*^−/−^ cells showed a 3.2 5hmdC-D3 vs 5.8 5hmdC per 10^4^ dG upon 10 μM 5hmdC-D3 exposure; 2.82 5hmdC-D3 vs 4.91 5hmdC per 10^4^ dG upon 20 μM 5hmdC-D3 exposure. Plots represent mean values from 3 biological replicates. **B** *Left*, representative immunofluorescence of *wild type* and *Fancd2*^−/−^ MEFs showing PAR nuclear staining and EdU positive (EdU+) cells after exposure to 5hmdC (10 μM) for 16 h (*n* = 3). *Right*, bar plot showing percentage of PAR+ cells in EdU negative (EdU−) or EdU+ cell populations (*n* = 250 of each of 3 independent biological replicates, Student’s *t* test; bars represents mean ± s.d.). **C** Western blot of iPOND-captured and input protein extracts from *wild type* and *Fancd2*^−/−^ cells treated with 5hmdC (160 μM) or HU (1 mM) for 3 h to detect γ-H2AX, PCNA and RAD51. **D** *Top left*, scheme of BrdU incorporation assay. *Bottom left*, representative immunofluorescence images of *wild type* and *Fancd2*^−/−^ MEFs showing BrdU + (*Red*) or BrdU- cells and nuclear DNA (DAPI, *blue*). *Right*, percentage of BrdU+ or BrdU− cells after HU (1 mM) or 5hmdC (40 μM) for 16 h (*n* = 200 of each of 3 biological replicates, Student’s *t* test; Bar represents mean ± s.d.). **E** *Top*, plot depicting PAR mean intensity signal per nucleus of *wild type* and *Fancd2*^−/−^ cells exposed to 10, 40, 80 or 160 μM 5hmdC for 1 h (*n* = 3, Mann–Whitney test; central line represents median value). *Bottom*, plot depicting γ-H2AX foci per nucleus of *wild type* and *Fancd2*^−/−^ cells exposed to 10, 40, 80 or 160 μM 5hmdC for 1 h (*n* = 3, Student’s *t* test; central line represents mean ± s.d.). **F** *Top left*, scheme of the DNA fiber assay. *Bottom left*, representative images of DNA fibers from *wild type* and *Fancd2*^−/−^ MEFs after 10, 40, 80 or 160 μM 5hmdC exposure. *Right*, plot representing fork speed (Kbp min^−1^) of *wild type* and *Fancd2*^−/−^ MEFs exposed to 10, 40, 80 or 160 μM 5hmdC (*n* = 150 of each of 3 biological replicates, Mann–Whitney test; central line represents median value).

To gain further insight into how 5hmdC induced genotoxicity of FA cells, we sought to investigate replication fork dynamics. Firstly, 5hmdC genotoxicity was unrelated to release of any aldehyde breakdown product (Supplementary Fig. 4A), but suppressed by supplementation with free unmodified DNA bases (Supplementary Fig. 4B). We then reasoned that 5hmdC genotoxicity may arise from its incorporation during DNA synthesis. To examine 5hmdC incorporation into genomic DNA, we made use of isotopically labeled 5hmdC (5hmdC-D3), and determined the abundance of both naturally occurring and deuterated isotopologues of 5hmdC by HPLC-MS/MS (Supplementary Fig. 5A, B), which allows a direct comparison of exogenous versus endogenous genomic 5hmdC. We found that 5hmdC-D3 accumulated in genomic DNA in a cell line and dose dependent manner (Fig. 2A). *Wild type* cells fed with 5hmdC-D3 (10 and 20 μM) incorporated levels around 10% and 20% of the endogenous 5hmdC, respectively, whereas the incorporation reached around 60% and 58% of the endogenous 5hmdC level in *Fancd2*^−/−^ cells, (Fig. 2A), presumably explaining the observed sensitivity.

Consistent with the involvement of PARP1 during removal of 5hmdC during BER, 5hmdC treatment readily increased nuclear PARylation mainly in EdU positive cells at larger extent in FANCD2 deficient cells (Fig. 2B), probably reflecting the presence of DNA damage associated to 5hmdC incorporation during DNA replication in the absence of a functional FA pathway. Moreover, iPOND pulldowns of EdU-associated replication forks efficiently coprecipitated γ-H2AX from 5hmdC-treated *Fancd2*^*−/−*^ cells (Fig. 2C), suggesting that 5hmdC-induced γ-H2AX foci associated to stalled replication forks. In fact, 5hmdC incorporation during DNA synthesis attenuated subsequent BrdU incorporation in *Fancd2*^−/−^ cells (≈56% BrdU+ cells untreated vs ≈22.8% 5hmdC condition) (Fig. 2D). Because incorporation of hydroxymethylated DNA bases may hinder DNA synthesis, we examined how 5hmdC incorporation affected replication fork dynamics. We firstly determined the precise dose of 5hmdC to be used by examining nuclear PARylation and γ-H2AX foci formation. Whereas PARylation was significantly heightened in *Fancd2*^−/−^ cells at 10 μM 5hmdC, γ-H2AX foci formation reached significance at 40 μM (Fig. 2E). We matched the experimental settings described in Fig. 2E, and increased IdU supplementation period to 60 min. 5hmdC supplementation severely impaired fork speed in *Fancd2*^−/−^ cells (untreated, 1.24 Kbp min^−1^; 10 μM, 1.08 Kbp min^−1^; 40 μM, 1.04 Kbp min^−1^; 80 μM, 0.75 Kbp min^−1^; 160 μM, 0.64 Kbp min^−1^) whereas in *wild type* cells remained relatively unchanged (untreated, 1.268 Kbp min^−1^; 10 μM, 1.27 Kbp min^−1^; 40 μM, 1.28 Kbp min^−1^; 80 μM, 1.26 Kbp min^−1^; 160 μM, 1.29 Kbp min^−1^) (Fig. 2F), demonstrating that 5hmdC incorporation impaired fork progression in the absence of a functional FA/BRCA pathway. In agreement with a role of FANCD2 during replication fork restart [38], 5hmdC exposure also increased the frequency of collapsed forks in *Fancd2*^−/−^ cells compared to untreated conditions (*Fancd2*^−/−^ Untreated:0.75 median IdU/CldU ratio; *Fancd2*^−/−^ +5hmdC: 0.5 median IdU/CldU ratio) (Supplementary Fig. 6), suggesting that 5hmdC-stalled replication forks restarted poorly in the absence of FANCD2. Taken together, these data suggest that 5hmdC is uptaken by cells, incorporated during DNA synthesis and affects replication dynamics by hindering fork progression, which necessitates FANCD2 to preserve fork stability.

### PARP1 trapping exacerbates replication fork defects of *Fancd2*^*−/−*^ cells upon 5hmdC exposure

**Fig. 3:**
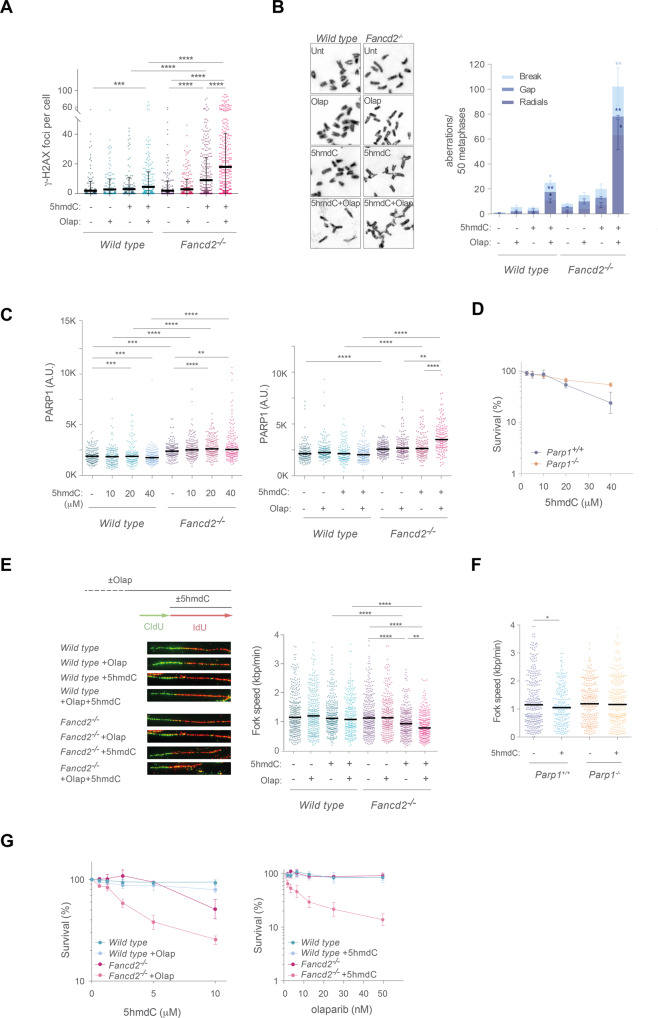
PARP1 is responsible for 5hmdC-induced replication fork instability in *Fancd2*^−/−^ cells. **A** Dot plot showing quantitation of nuclear γ-H2AX foci in *wild type* and *Fancd2*^−/−^ MEFs upon treatment with 5hmdC (2 μM), olaparib (7.5 nM) or 5hmdC+olaparib for 16 h (*n* = 100 of each of 3 biological replicates, Student’s *t* test; central line represents mean ± s.d.). **B** *Left*, representative images of chromosome metaphases from *wild type* and *Fancd2*^−/−^ cells treated with 5hmdC (2 μM), olaparib (7.5 nM) or 5hmdC+olaparib. *Right*, bar plot of breakdown of the different types of chromosomal aberrations (*n* = 150 of each of 3 biological replicates, Student’s *t* test; bar represents mean ± s.d.). **C** Dot plot representing immunostaining of PARP1 during the chromatin retention assay from *wild type* and *Fancd2*^−/−^ cells exposed to 5hmdC (10, 20, 40 μM) for 3 h (*left*) or in combination with olaparib (0.1 μM + 10 μM 5hmdC) (*right*). Olaparib was added 30 min before 5hmdC (*n* = 3, Mann–Whitney test; central line represents median value). **D** Cell proliferation assay of *Parp1*^+/+^ and *Parp1*^−/−^ MEFs exposed to the indicated doses of 5hmdC for 4 days (*n* = 4, mean ± s.d.). **E** *Top left*, scheme of the DNA fiber assay of *wild type* and *Fancd2*^−/−^ cells in the presence of 5hmdC in combination with olaparib. *Bottom left*, representative images of DNA fibers from *wild type* and *Fancd2*^−/−^ MEFs after 5hmdC (40 μM) exposure alone or in combination with olaparib (0.5 μM). *Right*, plot representing fork speed (Kbp min^−1^) (*n* = 150 of each of 3 biological replicates, Mann–Whitney test; central line represents median value). **F** Dot plot representing fork speed (Kbp min^−1^) of *Parp1*^+/+^ and *Parp1*^−/−^ in the presence of 5hmdC (40 μM)(*n* = 150 of each of 3 biological replicates, Mann–Whitney test; central line represents median value). **G** *Left*, cell proliferation assay of *wild type* and *Fancd2*^−/−^ MEFs exposed to the indicated doses of 5hmdC alone or combined with a fixed dose of olaparib (15 nM), for 3 days (*n* = 4, mean ± s.d.), or *right*, to the indicated doses of olaparib alone or combined with a fixed dose of 5hmdC (1.25 μM) for 3 days (*n* = 4, mean ± s.d.).

During removal of alkylated DNA bases by BER, PARP1 facilitates DNA repair by attracting DNA repair proteins to DNA breaks that arise from APE1-mediated lyase activity [39]. PARP inhibitors are currently being used in the clinic to treat several HR-deficient tumors, which leads to synthetic enhancement of cell toxicity owing to fork collapse at trapped-PARP1 on BER intermediates or genomic ribonucleotides [40–43]. We therefore used low dose of 5hmdC in combination with olaparib to study the synergistic effect of PARP1 trapping in the absence of a functional FA/BRCA pathway. PARP1 inhibition significantly increased 5hmdC-dependent γ-H2AX in *Fancd2*^−/−^ cells compared to single treatments (Fig. 3A), and to a lesser extent in *wild type* cells. PARP1 trapping also increased the frequency of chromosomal aberrations in *Fancd2*^−/−^ cells (Fig. 3B), indicating that trapped PARP1 during BER intermediates at 5hmdC lesions gave rise to DSBs, which accounted for the increased frequency of chromosome aberrations. PARP1 recruitment to chromatin also increased in the absence of FANCD2, suggesting the increased presence of endogenous DNA damage in FA cells. Moreover, PARP1 chromatin recruitment by 5hmdC was dose dependent in the absence of FANCD2 (Fig. 3C and Supplementary Fig. 7), and exacerbated by olaparib treatment (Fig. 3C) suggesting that 5hmdC removal from DNA by BER led to PARP1 chromatin recruitment, which was significantly increased in the absence of FANCD2 and exacerbated by olaparib, although PARP1 overexpression did not enhance 5hmdC-dependent DNA damage (Supplementary Fig. 8). Additionally, *Parp1*^−/−^ cells were not only insensitive but if so, resistant to 5hmdC compared to *Parp1*^+/+^ cells (Fig. 3D), suggesting that trapped PARP1 complexes accounted for the noticeable lethality seen in *Parp1*^+/+^ cells. To examine the effect of trapped-PARP1 complexes by olaparib at 5hmdC DNA lesions on replication fork dynamics, we used 40 μM 5hmdC, a dose sufficient to cause a marked defect in replication fork in *Fancd2*^−/−^ cells (Fig. 2F). Despite of the lack of impairment on fork speed in *wild* type cells, olaparib largely exacerbated 5hmdC-mediated fork defects observed in *Fancd2*^−/−^ cells (Fork speed unt 1.10 Kbp/min; 1.11 Kbp/min Olap; 0.90 Kbp/min 5hmdC; 0.76 Kbp/min 5hmdC+Olap) (Fig. 3E), suggesting that FANCD2 safeguards the integrity of replication forks at 5hmdC sites of damage, presumably due to PARP1-replication conflicts. In agreement with this, 5hmdC exposure unveiled a fork defect in *Parp1*^+/+^ *wild type* cells compared to *Parp1*^−/−^ (Fork speed *Parp1*^+/+^ unt 1.27 Kbp/min *Parp1*^+/+^ 5hmdC 0.96 Kbp/min; *Parp1*^−/−^ unt 1.22 Kbp/min; *Parp1*^−/−^ 5hmdC 1.24 Kbp/min) (Fig. 3F), suggesting that PARP1 contributes to replication fork defects during the processing of 5hmdC. As a consequence, combination of a fixed low dose of olaparib with increasing doses of 5hmdC additively increased *Fancd2*^−/−^ cell lethality (Fig. 3G). Conversely, a fixed dose of 5hmdC also caused an olaparib dose-dependent death of *Fancd2*^−/−^ cells (Fig. 3G), indicating that the persistence of PARP1 in chromatin is responsible for the elevated accumulation of replication fork defects, chromosomal aberrations and sensitivity in the absence of FANCD2.

### 5hmdC is converted to 5hmdU in vivo

**Fig. 4:**
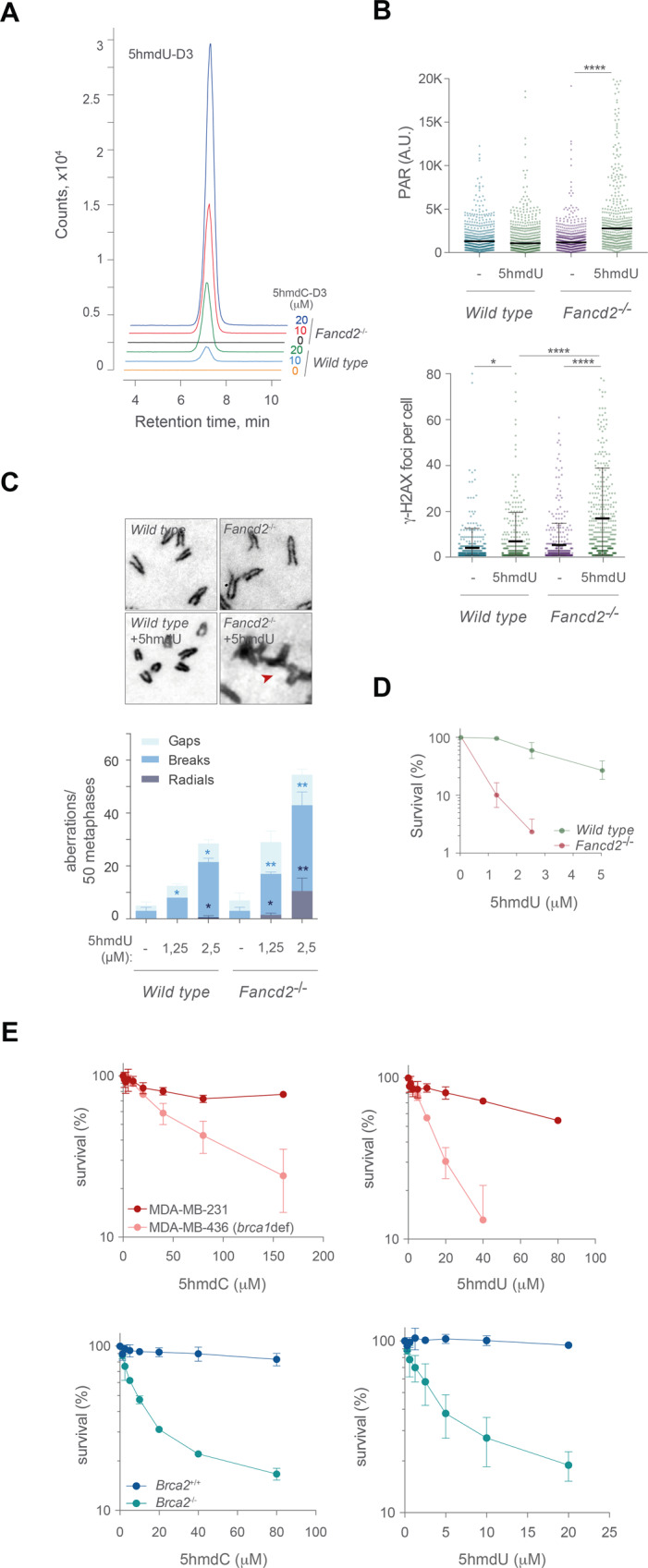
5hmdC derived 5hmdU is responsible for the DNA damage and genomic instability in *Fancd2*^−/−^ cells. **A** Plot representing the quantitation by HPLC-MS/MS of exogenous 5hmdU-D3 level in genomic DNA samples from *wild type* and *Fancd2*^−/−^ cells exposed to isotopically labeled 5hmdC-D3 (0,10 and 20 μM) for 16 h. **B** *Top*, plot depicting PAR mean intensity signal per nucleus of *wild type* and *Fancd2*^−/−^ cells exposed to 5hmdU (2.5 μM) for 16 h (*n* = 3, Mann–Whitney test; central line represents median value). *Bottom*, plot depicting γ-H2AX foci obtained from immunofluorescence images from *wild type* or *Fancd2*^−/−^ cells exposed to 5hmdU (2.5 μM) for 16 h (*n* = 3, Student’s *t* test; central line represents mean ± s.d.). **C** *Top*, representative images of chromosome aberrations (red arrowhead) test from *wild type* and *Fancd2*^−/−^ cells following 5hmdU treatment (1.25 and 2.5 μM) for 40 h. *Bottom*, bar plot of breakdown of the different types of chromosomal aberrations (*n* = 150 of each of 3 biological replicates, Student’s *t* test; bar represents mean ± s.d.). **D** Cell proliferation assay of *wild type* and *Fancd2*^−/−^ MEFs exposed to the indicated doses of 5hmdU for 3 days (*n* = 4, mean ± s.d.). **E** Cell proliferation assays of MDA-MB-231 (BRCA1 proficient), MDA-MB-436 (BRCA1 deficient), BRCA2^+/+^ and BRCA2^−/−^ DLD-1 cell exposed to the indicated doses of 5hmdC or 5hmdU for 7 days (*n* = 4, mean ± s.d).

As mentioned before, *Fancd2*^−/−^ cells exposed to 5hmdC-D3 presented ∼3 5hmdC-D3 molecules per 10^6^ dG (Fig. 2A). Deep examination of chromatogram peaks also showed the presence of 5hmdU-D3 on genomic DNA, not detected on untreated cells. This result was quite surprising and indicated that 5hmdC-D3 was converted into 5hmdU-D3. Similar to 5hmdC-D3, 5hmdU-D3 on genomic DNA also accumulated in a dose- and cell line-dependent manner (Fig. 4A and Supplementary Fig. 5). The accumulation of genomic 5hmdU-D3 in *Fancd2*^−/−^ was ~4-5 fold higher than in *wild type* cells (*wild type* 0.12% 5hmdU-D3/dG, *Fancd2*^−/−^ 0.62% 5hmdU-D3/dG for 10 μM 5hmdC-D3; *wild type* 0.64% 5hmdU-D3/dG, *Fancd2*^−/−^ 2.22% 5hmdU-D3/dG for 20 μM 5hmdC-D3). More strikingly, 5hmdC-D3 conversion to 5hmdU-D3 resulted in ~2 × 10^3^-fold increase of 5hmdU-D3 over 5hmdC-D3 (Fig. 4A). These results suggest that 5hmdU-D3 derived from 5hmdC-D3 is presumably the most toxic incorporated 5-hydroxymethylated nucleotide to *Fancd2*^−/−^ cells.

Examination of the DNA damage by 5hmdU showed that 5hmdU exposure led to a marked increase of PARylation in *Fancd2*^−/−^ cells not detected in *wild type* cells (Fig. 4B). 5hmdU exposure also led to accumulation of γ-H2AX (Fig. 4B) suggesting that 5hmdU activated PARP enzymes presumably at SSBs generated by the concerted action of the SMUG1 and APE1 nucleases [44]. Similar to 5hmdC, 5hmdU exposure significantly increased the frequency of chromosomal aberrations in *Fancd2*^−/−^ cells (Fig. 4C), suggesting that processing of 5hmdU likewise 5hmdC, generates SSBs that are converted into DSB and induces genomic instability. *Fancd2*^−/−^ cells were more sensitive to 5hmdU than *wild type* cells (Fig. 4D), probably as a consequence of increased genomic instability and DNA damage inflicted by 5hmdU incorporation. Moreover, BRCA1 (MDA-MB-436) or BRCA2 (*BRCA2*^−/−^ DLD-1) cell lines also showed 5hmdC and 5hmdU sensitivity in comparison to their *wild type* counterparts (Fig. 4E), thus extending these findings to other components of the FA/BRCA pathway.

### 5hmdU blocks replication fork progression and traps PARP1 on chromatin

**Fig. 5:**
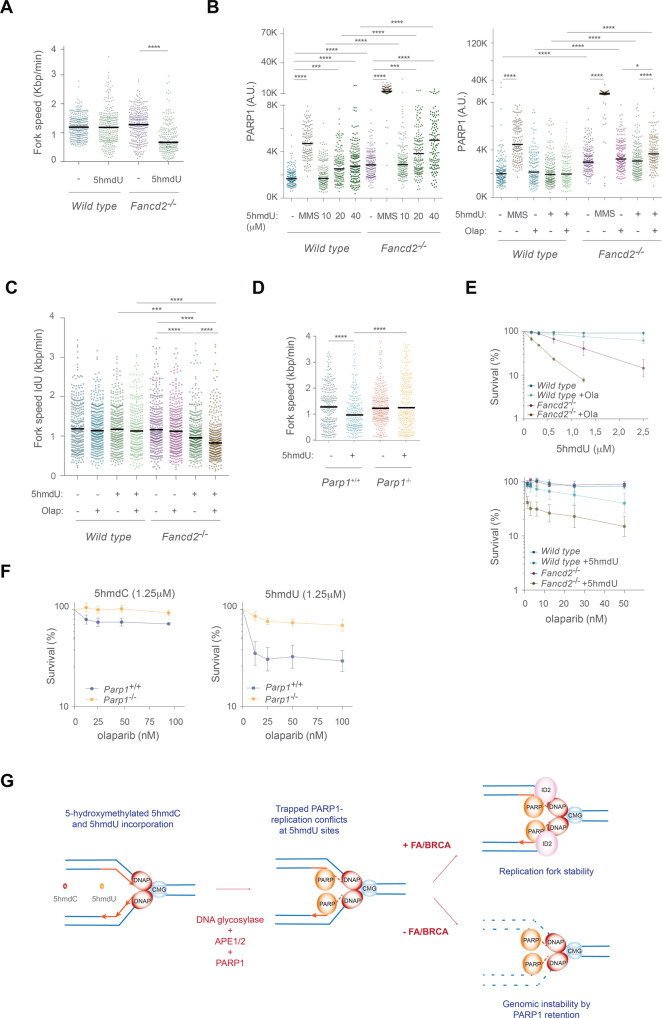
PARP1 trapping by olaparib is responsible for 5hmdU induced replication fork instability in *Fancd2*^−/−^ cells. **A** Dot plot representing fork speed (Kbp min^−1^) of *wild type* and *Fancd2*^−/−^ cells exposed to 5hmdU (40 μM) (*n* = 150 of each of 3 biological replicates, Mann–Whitney test; central line represents median value). **B** *Left*, dot plot representing the PARP1 chromatin retention assay from *wild type* and *Fancd2*^−/−^ cells upon exposure to 5hmdU (0, 10, 20 and 40 μM) for 3 h. *Right*, PARP1 chromatin retention assay of *wild type* and *Fancd2*^−/−^ cells exposed to 5hmdU (10 μm), olaparib (0.1 μM) or combination of 5hmdU + olaparib for 3 h. **C** Dot plot representing replication fork speed of *wild type* and *Fancd2*^−/−^ cells exposed to 5hmdU (40 μM), olaparib (0.5 μM) or a combination of 5hmdU + Olaparib. Olaparib was added 60 min before adding CldU (*n* = 150 of each of 3 biological replicates, Mann–Whitney test; central line represents median value). **D** Dot plot representing replication fork speed of *Parp1*^+/+^ and *Parp1*^−/−^ cells exposed to 5hmdU (40 μM) (*n* = 150 of each of 3 biological replicates, Mann–Whitney test; central line represents median value). **E** *Top*, cell proliferation assay of *wild type* and *Fancd2*^−/−^ MEFs exposed to the indicated doses of 5hmdU alone or combined with a fixed dose of olaparib (15 nM) for 3 days (*n* = 4, mean ± s.d.). *Bottom*, cell proliferation assay of *wild type* and *Fancd2*^−/−^ MEFs exposed to the indicated doses of olaparib alone or combined with a fixed dose of 5hmdU (1.25 μM), for 3 days (*n* = 4, mean ± s.d.). **F** Cell proliferation assay of *Parp1*^+/+^ and *Parp1*^−/−^ MEFs exposed to the indicated doses of olaparib combined with a fixed dose (1.25 μM) of either 5hmdC (*left*) or 5hmdU (*right*) for 3 days (*n* = 4, mean ± s.d.). **G** Model depicting the instability of replication fork in the absence of FANCD2, caused by the recruitment of PARP1 to sites of 5hmdC or 5hmdU removal by the excision repair machinery.

We next examined whether 5hmdU exposure led to perturbation on the replication dynamics. Similar to 5hmdC, 5hmdU exposure significantly impacted on IdU fork speed (Fork speed *wild type* unt 1.19 Kbp min^−1^; *wild type* 5hmdU 1.18 Kbp min^−1^; *Fancd2*^−/−^ unt 1.28 Kbp min^−1^; *Fancd2*^−/−^ 5hmdU 0.66 Kbp min^−1^) (Fig. 5A), indicating that 5hmdU incorporation blocked replication fork progression. 5hmdU exposure also led to efficient chromatin recruitment of PARP1 in a dose dependent manner, which was further retained/trapped by olaparib (Fig. 5B) probably accounting for the replication fork defects seen in *Fancd2*^−/−^ cells by 5hmdU. Similarly, combination of 5hmdU and olaparib did not cause any replication defects in *wild type* cells, whereas this combination efficiently blocked replication fork progression in *Fancd2*^−/−^ cells (Fig. 5C) suggesting that during 5hmdU incorporation and processing by BER, PARP1 is strongly retained or trapped on chromatin, hindering replication fork progression. In agreement with this notion, 5hmdU exposure also impaired replication dynamics in *Parp1*^+/+^ cells compared to *Parp1*^−/−^ (Fig. 5D) indicating that the presence of PARP1 protein at sites of 5hmdU elimination during BER represent a major obstacle to the DNA replisome. Moreover, *Fancd2*^−/−^ cell lethality increased by either combination of a fixed dose of olaparib with increasing doses of 5hmdU, or by a fixed dose of 5hmdU combined with increasing dose of olaparib, indicating that PARP1 at sites of 5hmdU repair accounted for the replication defects and cell lethality seen in the absence of FANCD2 (Fig. 5E). Finally, olaparib exposure markedly affected the survival of *Parp1*^+/+^ cell in comparison to *Parp1*^−/−^ in the presence of a fixed dose of 5hmdU, whereas equal dose of 5hmdC had a lower impact on *Parp1*^+/+^ cell lethality (Fig. 5F), a phenotype not due to differential p53 response observed in *Parp1*^−/−^ cells (Supplementary Fig. 9). All together, these results suggest that 5hmdC is converted into 5hmdU and incorporated during DNA synthesis, followed by BER-dependent removal. During 5hmdU removal, PARP1 is trapped on DNA which hinders replication fork progression necessitating a functional FA/BRCA pathway to maintain replication fork integrity (Fig. 5G).

## Discussion

Despite of the exquisite role of FA at the protection of replication fork-structured DNA during ICL repair, the main cellular role for the FA pathway is the safeguard of the replisome integrity from disassembly, to avoid spurious DNA transactions. Here we uncover a role for the FA/BRCA pathway at maintaining replication fork stability during misincorporation of the 5-hydroxymethylated bases 5hmdC and 5hmdU, two epigenetically modified DNA bases arising from the cytosine demethylation pathway that potentially can contaminate the cellular nucleoside pool. By feeding cells with these cytidine analog bases that naturally occur during the cytosine demethylation reactions, we mimicked the contamination of the nucleotide pool and therefore examined its contribution to genome instability. We found that 5hmdC was readily incorporated into genomic DNA, reaching up to 60% of the endogenous 5hmdC in the absence of a functional FA pathway, which ultimately led to extensive DNA damage and increased frequency of chromosomal instability. The damage was presumably due to impaired replication fork progression and cell cycle arrest. A deeper examination of nucleotide composition present in the genomic DNA samples showed a dramatic ∼2000-fold accumulation of 5hmdC-derived 5hmdU, the cytidine deamination byproduct of 5hmdC. Similar to 5hmdC exposure, 5hmdU led to almost identical phenotypes in *Fancd2*^−/−^ cells, suggesting that 5hmdU most certainly mediates the cytotoxic effects seen in FANCD2 deficient cells upon 5hmdC exposure. Although 5hmdU is normally generated by TET-mediated oxidation of thymine and to a lesser extent by 5hmdC deamination [45], a recent paper by Fugger et al. [44] found out that HR deficient cells in the presence of olaparib showed a synthetic lethal interaction with increased 5hmdU in the nucleotide pool compartment. The source of 5hmdU was the epigenetic DNA base 5hmdC, as the reported synthetic lethal phenotype was abrogated by the loss of the cytosolic cytidine deaminase enzyme DCTD, whereas TET1/2 deletion had a minor contribution. Interestingly, the authors also identified DNPH1, which breaks down 5hmdUMP into dRP and 5hmdU in the free nucleotide pool, as a novel sensitizing gene, suggesting that 5hmdUMP is a major contaminating source of the nucleotide pool threating genome integrity of HR deficient cells. Our data, in agreement with Fugger et al. suggest that the FA/BRCA pathway and the sanitizing nucleotide pool enzymes serve as a two-tier protection mechanisms of replication fork stability against incorporation of 5-hydroxymethylated nucleotides.

Whereas the mechanism of 5hmdC-derived 5hmdU cytotoxicity has long been studied, the cytotoxic effect of its incorporation has mainly been ascribed to defects on the base excision repair machinery during base removal. These defective DNA structures were related to the presence of AP sites, the generation of unprotected SSBs, or unligated DNA ends [46]. In addition, our data indicate that excessive PARP1 retention or trapping on chromatin during excision of 5hmdU is also an important determinant of cell lethality in the absence of FANCD2. Consistently, trapped PARP1 at 5hmdU-excision sites exacerbated the DNA damage, the replication fork defects and the chromosomal instability, which ultimately led to cell lethality. Nevertheless, ablation of PARP1 did not showed any overt replication fork or survival defects upon 5hmdC or 5hmdU supplementation, suggesting that PARP1 trapped at repair sites accounted for 5hmdU toxicity at great extent. In agreement with our data, Fugger et al. demonstrated that 5hmdC-derived 5hmdU is removed by a BER mechanism involving SMUG1 glycosylase and PARP1, as ablation of these genes suppressed the 5hmdU-mediated cytotoxicity. Taken together, these data indicate that the formation of repair intermediates during base excision threatens the progression of the replisome throughout the persistence of trapped PARP1 and suggests that covalently bound or strongly retained protein-DNA complexes during DNA base excision repair, are extremely harmful DNA lesions perturbing replisome activity. In agreement with this notion, specific DPCs of repair proteins like HMCES, a factor that covalently binds to AP sites and shields them to avoid replisome collapse [47, 48], shows a synthetic lethal interaction with the cytidine deaminase activity of APOBEC3A [49]. However, the contribution of crosslinked HMCES at 5hmdU-derived abasic sites to the replication fork defects seen in FA deficient cells remains to be determined. In addition to increased replication fork speed [41], persistent SSB at either replication forks or during the formation of okazaki fragments [50], our results indicate that the synthetic lethal phenotype caused by olaparib in HR-deficient cells is largely due to trapped PARP1-replication conflicts (PRCs) during excision of 5-hydroxymethylated bases. The development of novel modified hydroxymethylated DNA bases in combination with olaparib and conventional chemotherapy in HR-deficient ovarian, breast or pancreatic cancers, could be a novel therapeutic approach to improve overall patient survival.

## Supplementary information

Supplementary figure legends

Supplementary figure 1

Supplementary figure 2

Supplementary figure 3

Supplementary figure 4

Supplementary figure 5

Supplementary figure 6

Supplementary figure 7

Supplementary figure 8

Supplementary figure 9

Supplementary figure 10

Supplementary figure 11

Supplementary figure 12

aj-checklist

## Data Availability

All cell lines, reagents and data generated during this study are included in this published article and its Supplementary Information files. Additional data are available from the corresponding author on reasonable request.
