# Supplementary material for: FANCD2 maintains replication fork stability during misincorporation of the DNA demethylation products 5-hydroxymethyl-2’-deoxycytidine and 5-hydroxymethyl-2’-deoxyuridine
Source: Cell Death Dis. 2022 May 27;13(5):503. doi: 10.1038/s41419-022-04952-0 (PMC9142498; doi:10.1038/s41419-022-04952-0)
Supplement: Supplementary file 1 — Supplementary figure legends [file 41419_2022_4952_MOESM1_ESM.docx]

**Supplementary Figures. Peña-Gómez et al.**

**Supplementary figure 1: Generation of FANCD2 KO eHAP cells and functional evaluation**

(A) *Top*, Clustal alignment to determine mutations introduced by CRISPR-Cas9 in FANCD2 clones (wild type eHAP; sgFD2-4.2; sgFD2-4.4; sgFD2-5.14). *Bottom*, western blot of *FANCD2* CRISPR eHAP clone extracts to detect the FANCD2 protein. (+) cisplatin (1 μg/ml) for 16 hours. (B) *Top,* clonogenic survival assay of wild type and FANCD2 knockout eHAP clones treated with cisplatin (0, 0.125, 0.250 μM) or 5hmdC (0, 5, 10 μM) for 5 days. *Bottom,* MTT survival assay of cisplatin treated FANCD2 KO clones (n=4, mean ± s.d.).

**Supplementary figure 2: Survival of FA- deficient cells upon exposure to cytidine analogues**

(A) Cell survival assay of *wild type*, *FANCC*^-^, *FANCF*^-/-^, *FANCL*^-/-^ and *FANCG*^-/-^ DT40 cell lines exposed to the indicated dose of 5dC, 5mdC and 5hmdC, for 3 days (n=3, mean ± s.d.). (B) Western blots of wild type DT40 (*top*) or eHAP (*bottom*) cell extracts to detect FANCD2 monoubiquitylation upon exposure to 5hmdC (0, 20 and 40 μM) for 16 hours.

**Supplementary figure 3: G2/M cell cycle arrest by 5hmdC in the presence of CHK1 inhibitors**

(A) Bar plot depicting the frequency of G1, S or G2 populations of *wild type* and *Fancd2*^-/-^ cells exposed to 5dC, 5mdC and 5hmdC (10 μM) for 16 h (n=3, student *t*-test; bar represents mean ± s.d.). (B) Representative FACS plots and bar plot depicting the frequency of G1, S or G2 populations of *wild type* and *Fancd2*^-/-^ cells exposed to 5dC, 5mdC and 5hmdC (10 μM) for 1, 4, 8 or 12 h (n=4, student *t*-test; bar represents mean ± s.d.). (C) Bar plot depicting the frequency of G1, S or G2 populations of *wild type* and *Fancd2*^-/-^ cells exposed to 5hmdC (100 μM) for 30 min, in combination with AZD7762 (10 nM) or UCN-01 (10 nM), and subsequently analyzed after 48 hours in culture. AZD7762 or UCN-01 were added during the last 24 h (n=3, student *t*-test; bar represents mean ± s.d.).

**Supplementary figure 4: 5hmdC-induced lethality in *Fancd2*^-/-^ cells it is not ameliorated by 2-mercaptoethanol supplementation**

(A) MTT survival assay of *wild type* and *Fancd2*^-/-^ MEFs treated with 5hmdC for 3 days in the presence of the aldehyde quencher 2-mercaptoethanol (0, 50, 100 μM) (n=3, mean ± s.d.). (B) MTT survival assay of *wild type* and *Fancd2*^-/-^ MEFs treated with 5hmdC for 3 days in the presence of EmbryoMax Nucleosides cocktail (4X) (n=3, mean ± s.d.).

**Supplementary figure 5: 5hmdC determination in genomic DNA from *wild type* or *Fancd2*^-/-^ upon exposure to 5hmdC-D3.**

(A) Regression curve calculated using increasing amounts of 5hmdC standard by HPLC-MS/MS. (B) HPLC-MS/MS analysis of 5hmdC (green line) and D3-5hmdC (blue line) in genomic DNA hydrolysates obtained after treatment of *wild type* and *Fancd2*^-/-^ cells with 5hmdC-D3 (0, 10, 20 μM) for 16 hours.

**Supplementary figure 6: 5hmdC affects replication fork restart in the absence of FANCD2.**

Scheme of replication restart setup by DNA fiber assay (*Top left*). Representative images of DNA fibers from *wild type* and *Fancd2*^-/-^ MEFs upon 5hmdC exposure (100 μM) (*Bottom left*). Line plot showing quantitation of frequency of IdU/CldU ratio per fiber upon 5hmdC exposure (*Right*) (n=120, of each of 3 biological replicates).

**Supplementary figure 7: PARP1 trapping by 5hmdC in the absence of FANCD2.**

Dot plot representing PARP1 during the chromatin retention assay from *wild type* and *Fancd2*^-/-^ cells exposed to 5hmdC (40 μM) for 1 h (n=3, Mann Whitney test; central line represents median value).

**Supplementary figure 8: Overexpression of MmPARP1 do not exacerbate 5hmdC-mediated DNA damage.**

(A) Western blots of cellular extracts from *wild type* and *Fancd2*^-/-^ MEFs transiently overexpressing Flag-MmPARP1. * marks non specific crossreacting band. LAMIN A/C is used as loading control. (B) Dot plot depicting PAR mean intensity signal per nucleus of MmPARP1 overexpressing *wild type* and *Fancd2*^-/-^ cells exposed to 5hmdC (10 μM) for 16 hours (n=2, Mann Whitney test; central line represents median value). (C) Dot plot depicting γ-H2AX foci obtained from immunofluorescence images from MmPARP1 overexpressing *wild type* or *Fancd2*^-/-^ cells exposed to 5hmdC (10 μM) for 16 hours (n=2, student *t*-test; central line represents mean ± s.d.).

**Supplementary figure 9: p53 and p21 response in *Parp1*^+/+^ and *Parp1*^-/-^ cells exposed to 5hmC and MMS**

(A) MTT survival assay of *Parp1*^+/+^ and *Parp1*^-/-^ MEFs treated with MMS for 3 days (n=3, mean ± s.d.). (B) Proliferation rate of *Parp1*^+/+^ and *Parp1*^-/-^ MEFs (n=5, mean ± s.d.). (C) *Left*, Western blots of *Parp1*^+/+^ and *Parp1*^-/-^ MEFs extracts showing total p53, ser15-p53, p21 and LAMIN levels. *Right*, western blots of *Parp1*^+/+^ and *Parp1*^-/-^ MEFs extracts showing total p53, ser15-p53, p21 and LAMIN A/C levels after treatment with 5hmC (40 μM) or MMS (0.1mM) for 1, 2 and 4 h.

**Supplementary figure 10: Data related to replication fork dynamics of *wild type*, *Fancd2*^-/-^, *Parp1*^+/+^ and *Parp1*^-/-^ cells exposed to 5hmC or 5hmU**

**Supplementary figure 11: Open microscope fields related to replication fork dynamics of *wild type*, *Fancd2*^-/-^, *Parp1*^+/+^ and *Parp1*^-/-^ cells exposed to 5hmC or 5hmU**

**Supplementary figure 12: Uncropped western blots**
