# Supplementary figures and images for: FANCD2 maintains replication fork stability during misincorporation of the DNA demethylation products 5-hydroxymethyl-2’-deoxycytidine and 5-hydroxymethyl-2’-deoxyuridine
Source: Cell Death Dis. 2022 May 27;13(5):503. doi: 10.1038/s41419-022-04952-0 (PMC9142498; doi:10.1038/s41419-022-04952-0)

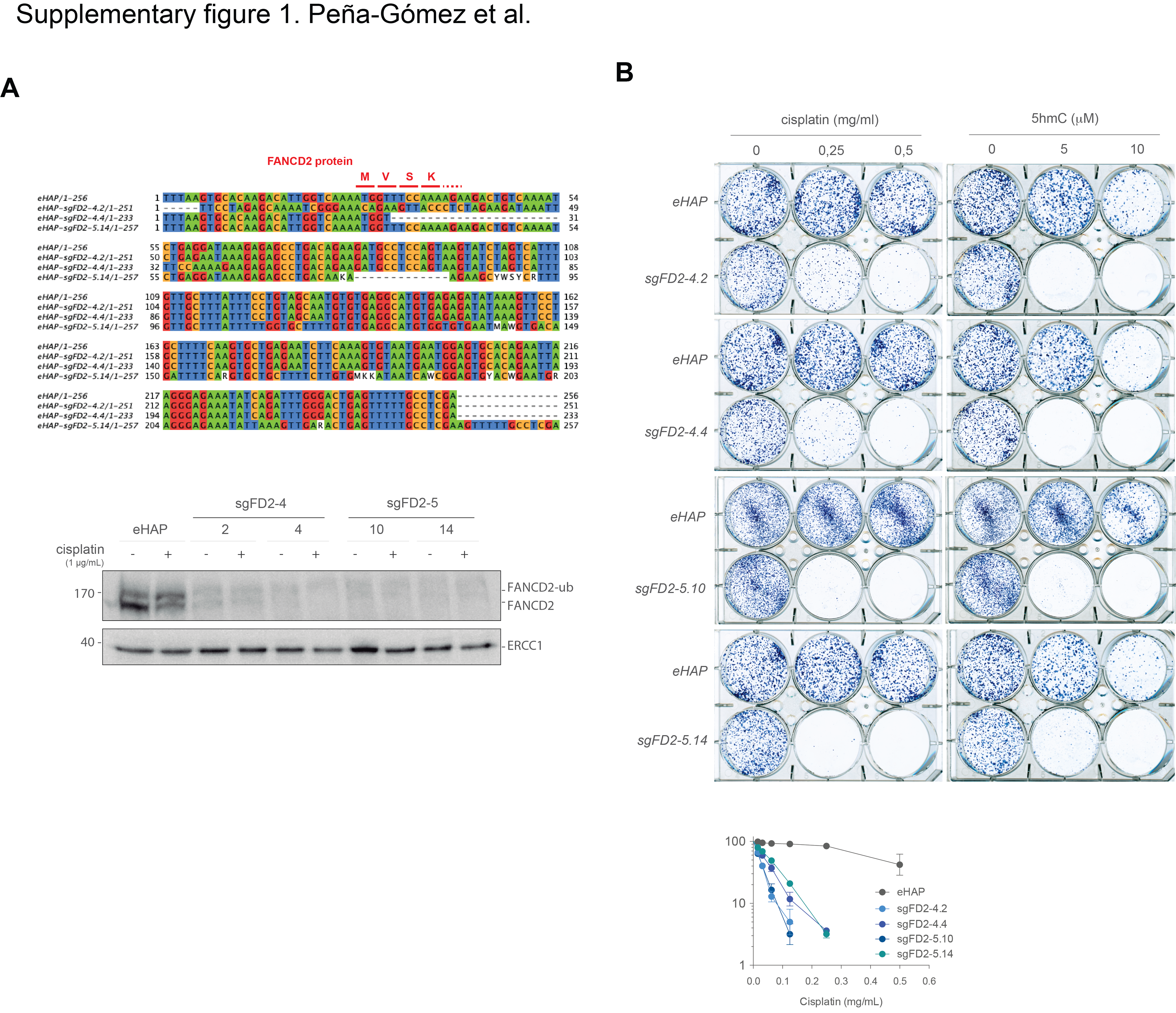

Supplement: Supplementary file 2 — Supplementary figure 1 [file 41419_2022_4952_MOESM2_ESM.png]

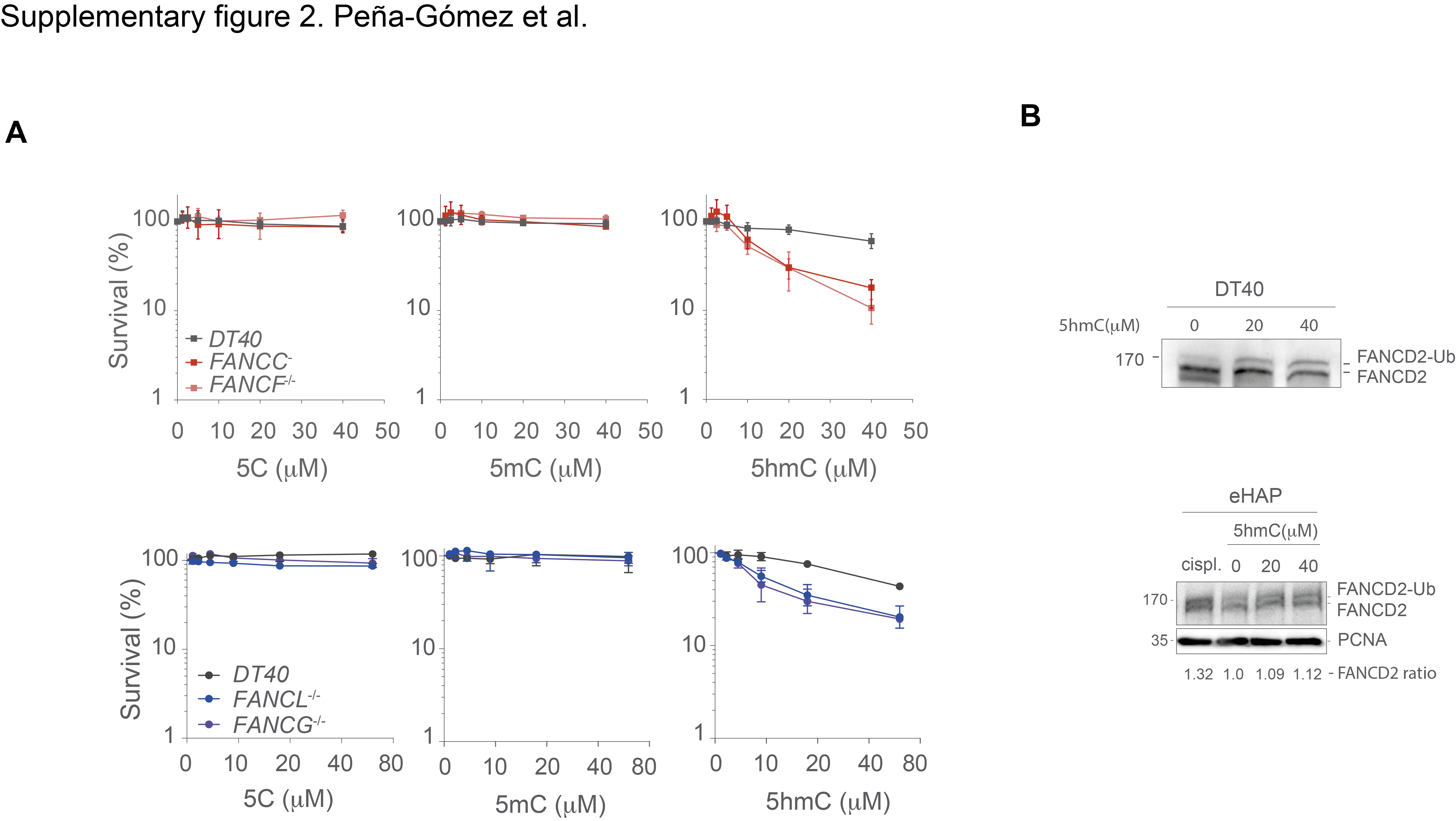

Supplement: Supplementary file 3 — Supplementary figure 2 [file 41419_2022_4952_MOESM3_ESM.png]

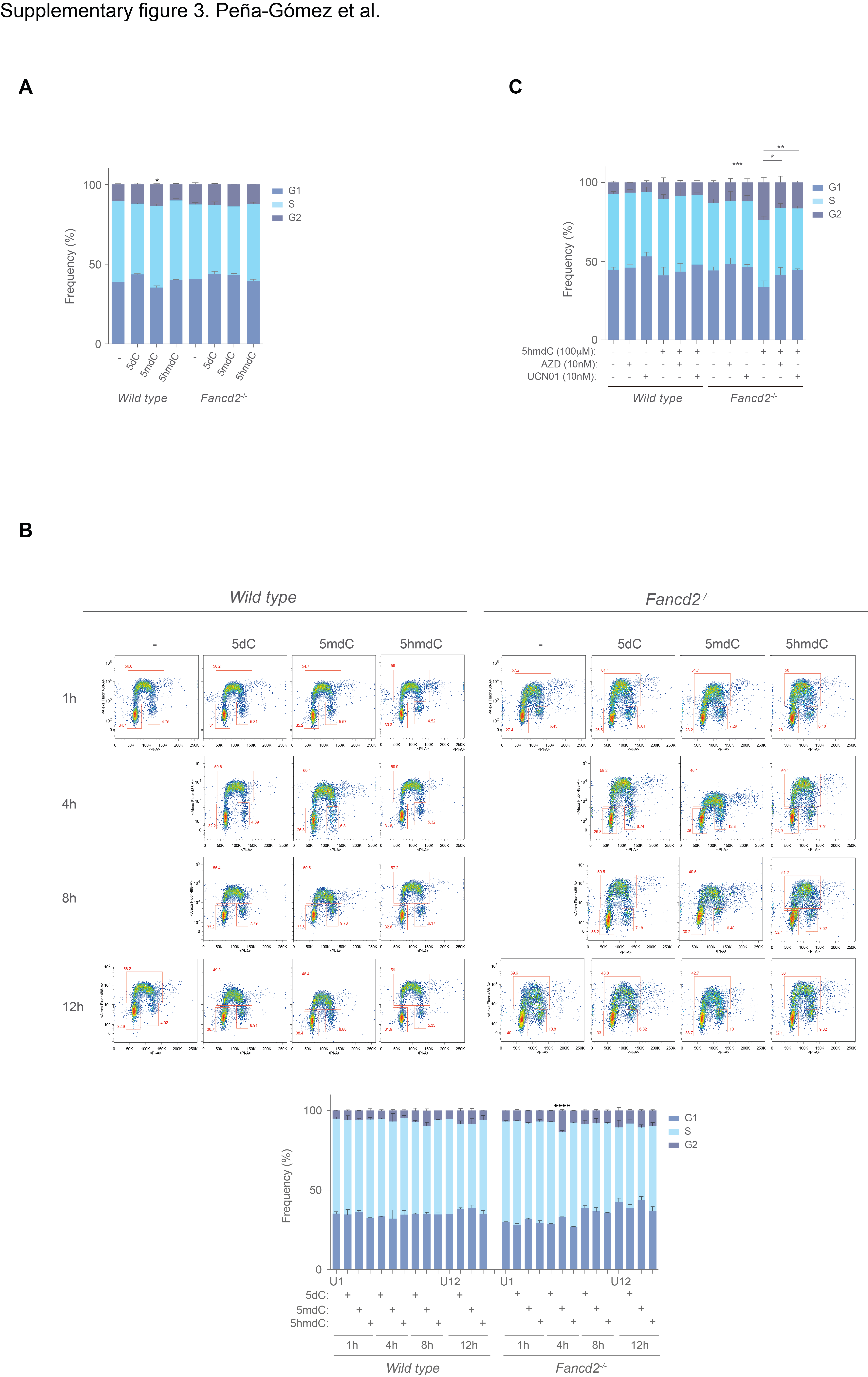

Supplement: Supplementary file 4 — Supplementary figure 3 [file 41419_2022_4952_MOESM4_ESM.png]

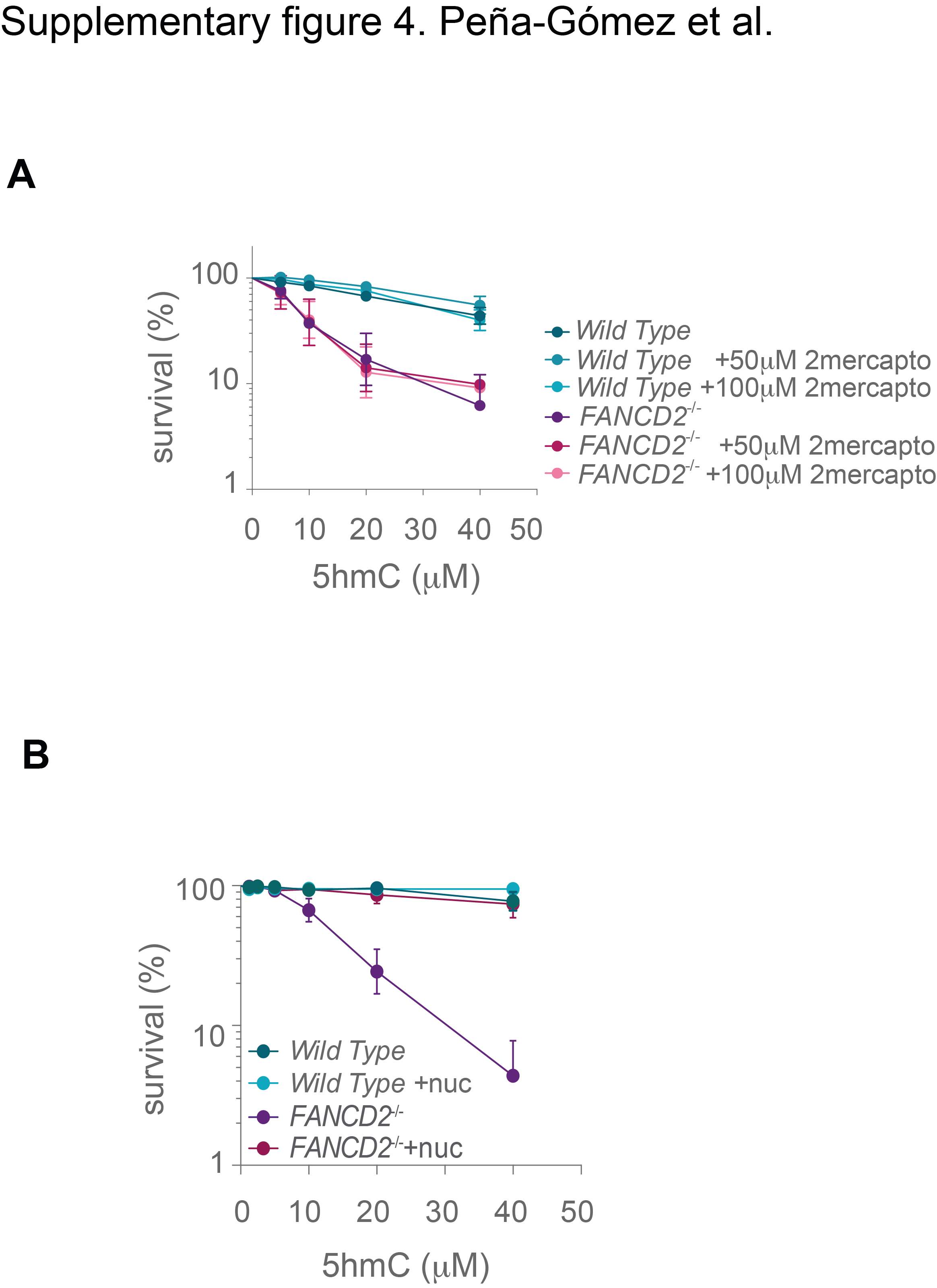

Supplement: Supplementary file 5 — Supplementary figure 4 [file 41419_2022_4952_MOESM5_ESM.png]

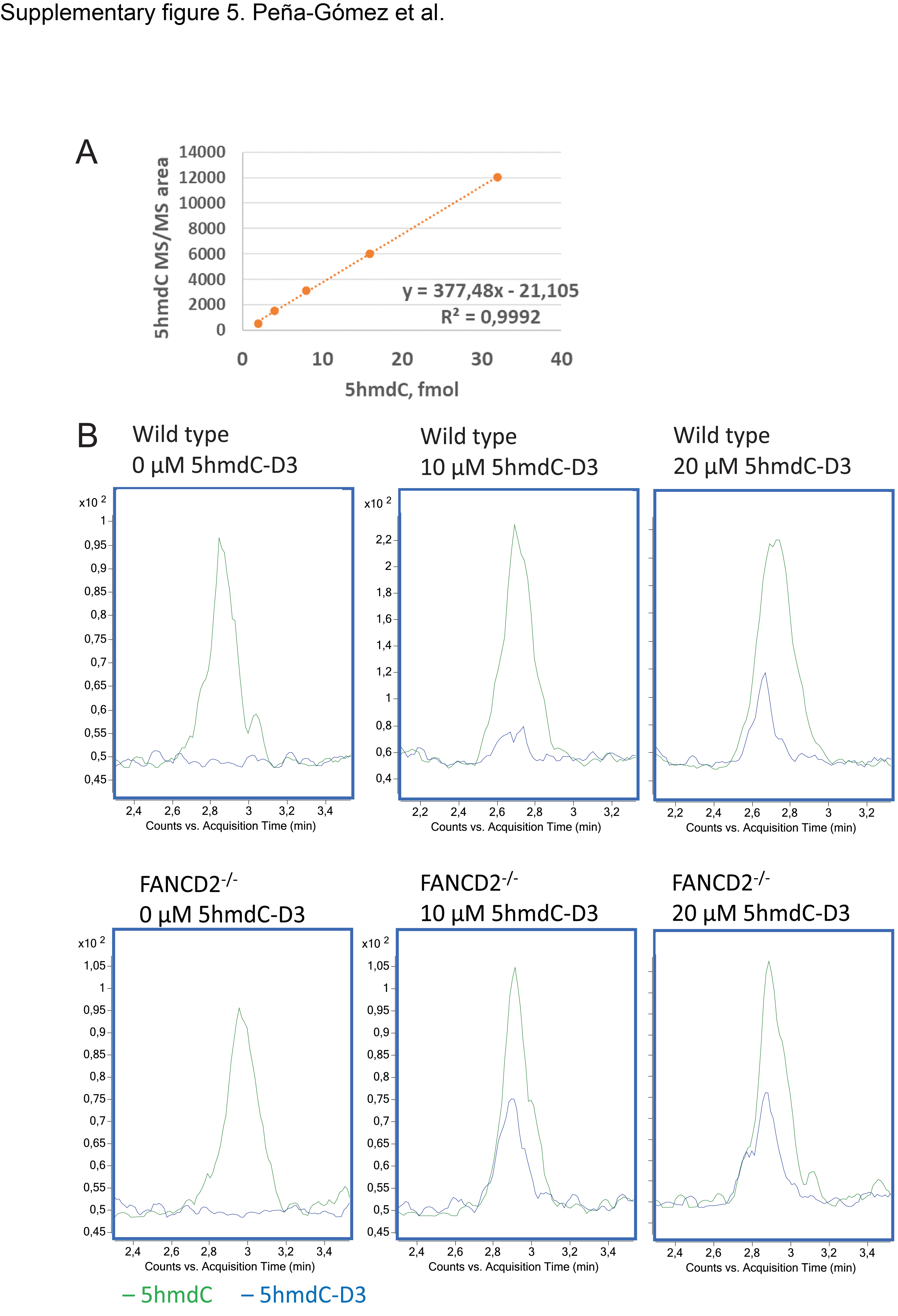

Supplement: Supplementary file 6 — Supplementary figure 5 [file 41419_2022_4952_MOESM6_ESM.png]

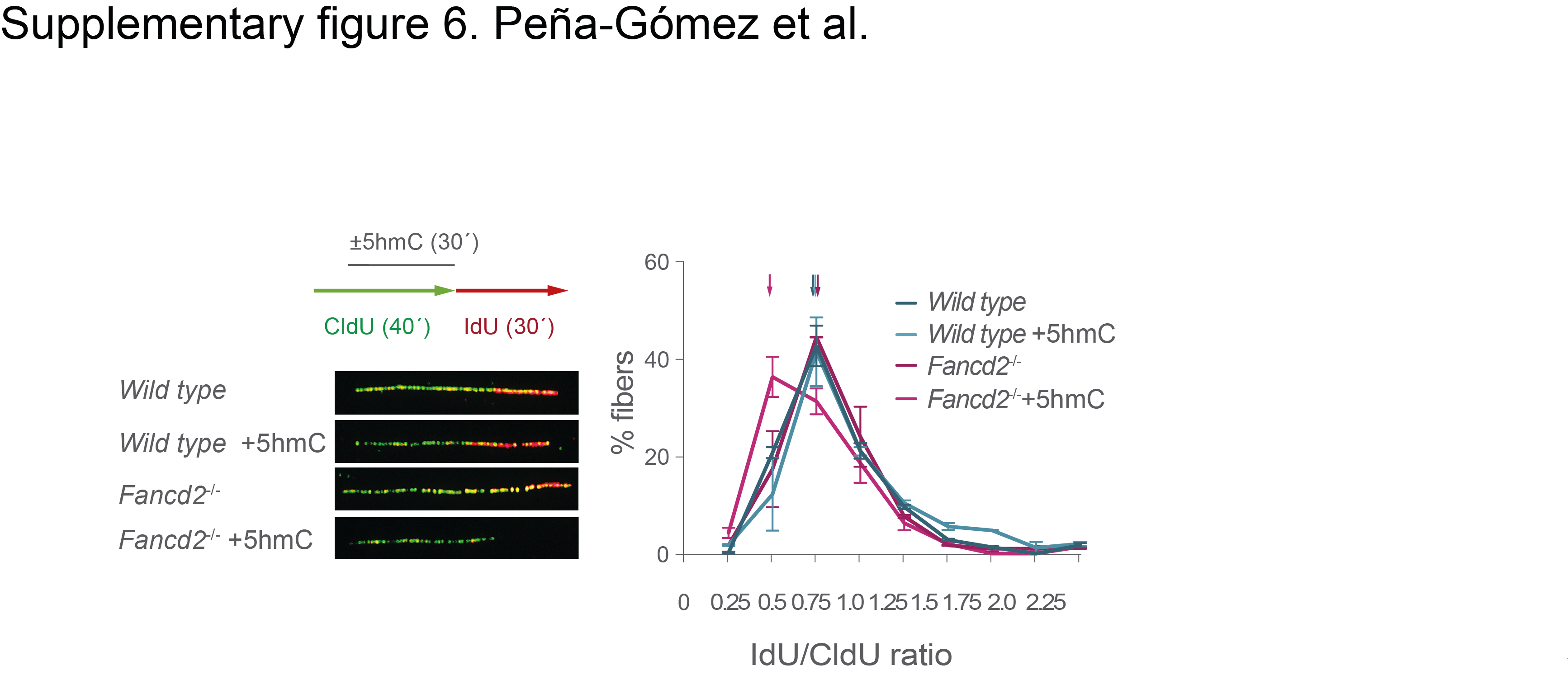

Supplement: Supplementary file 7 — Supplementary figure 6 [file 41419_2022_4952_MOESM7_ESM.png]

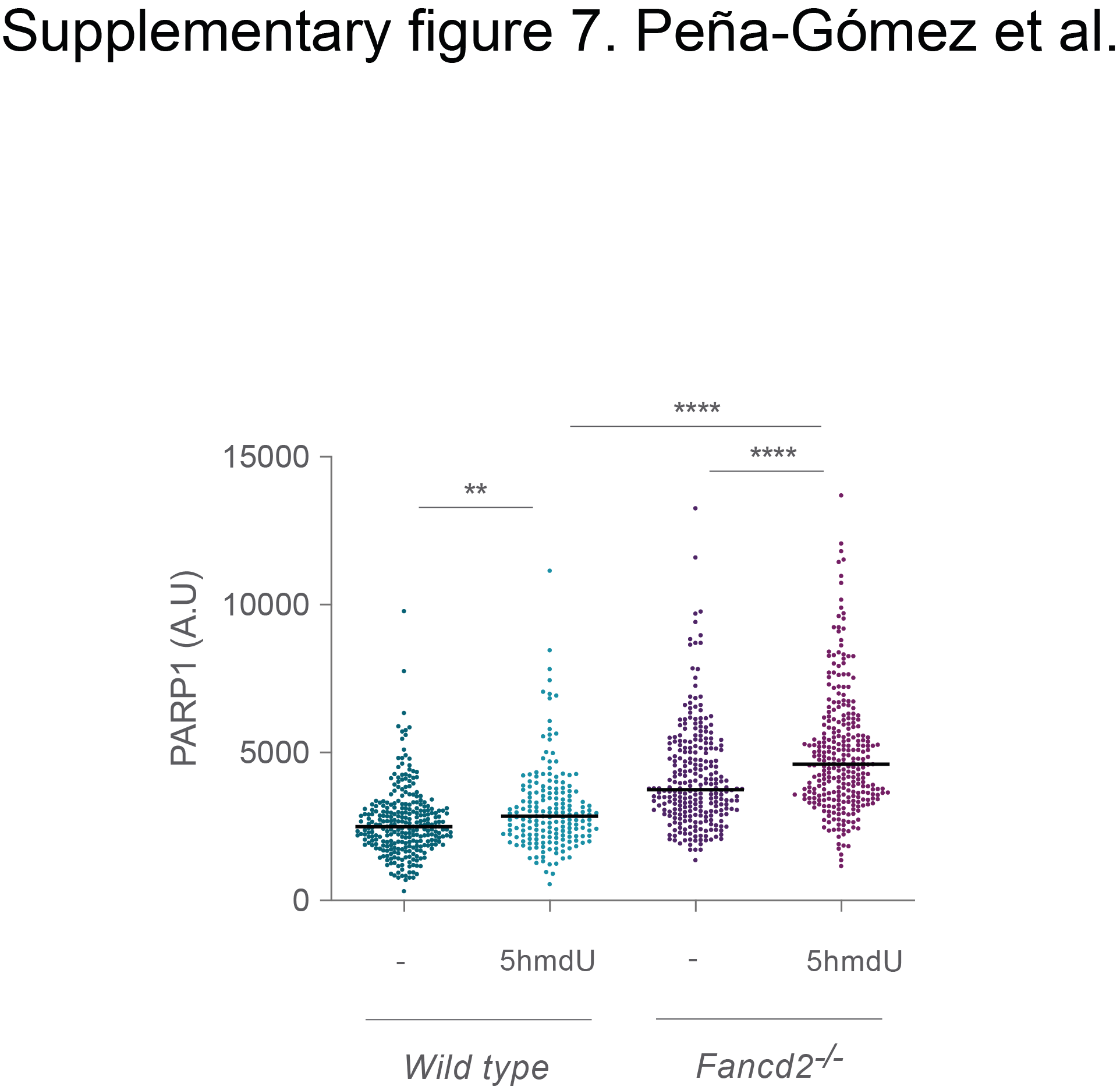

Supplement: Supplementary file 8 — Supplementary figure 7 [file 41419_2022_4952_MOESM8_ESM.png]

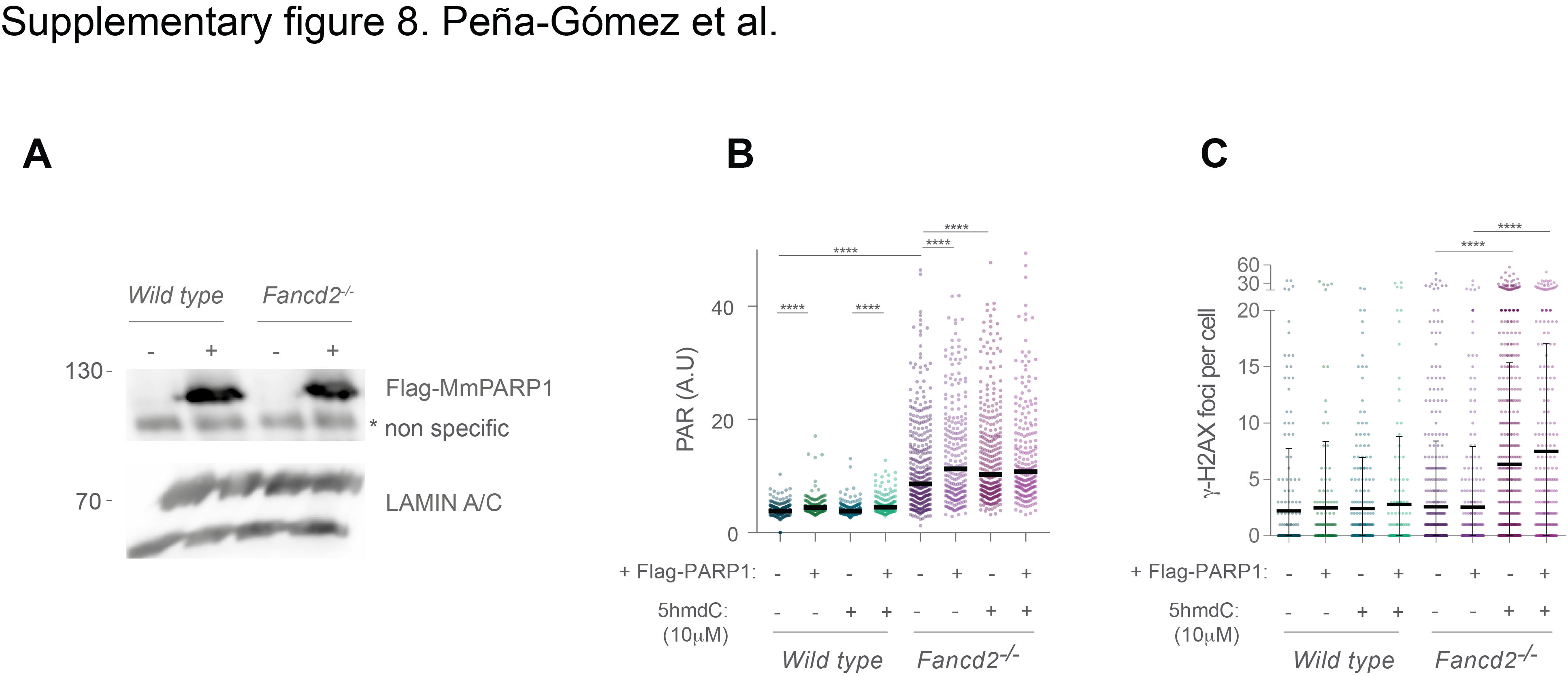

Supplement: Supplementary file 9 — Supplementary figure 8 [file 41419_2022_4952_MOESM9_ESM.png]

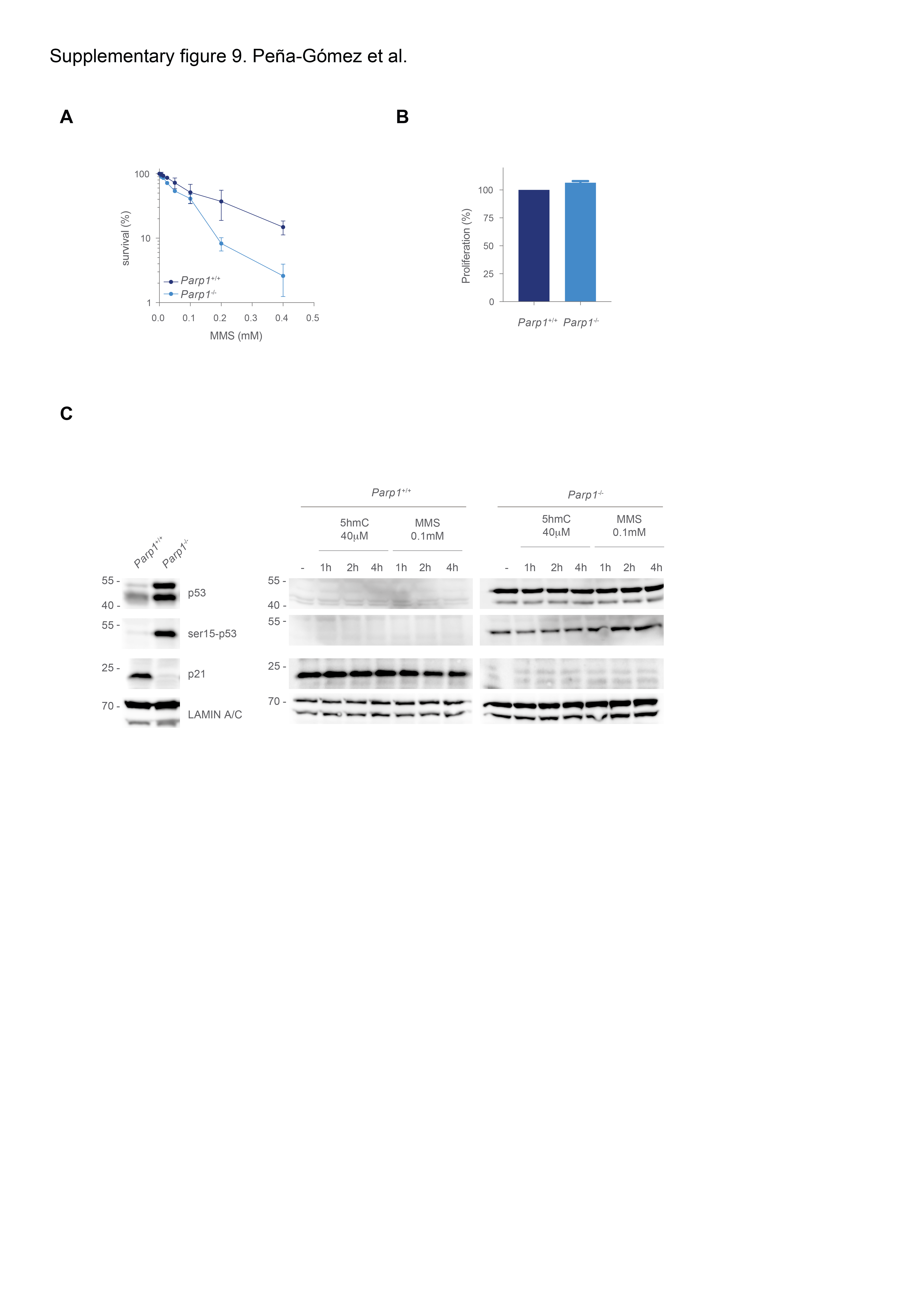

Supplement: Supplementary file 10 — Supplementary figure 9 [file 41419_2022_4952_MOESM10_ESM.png]

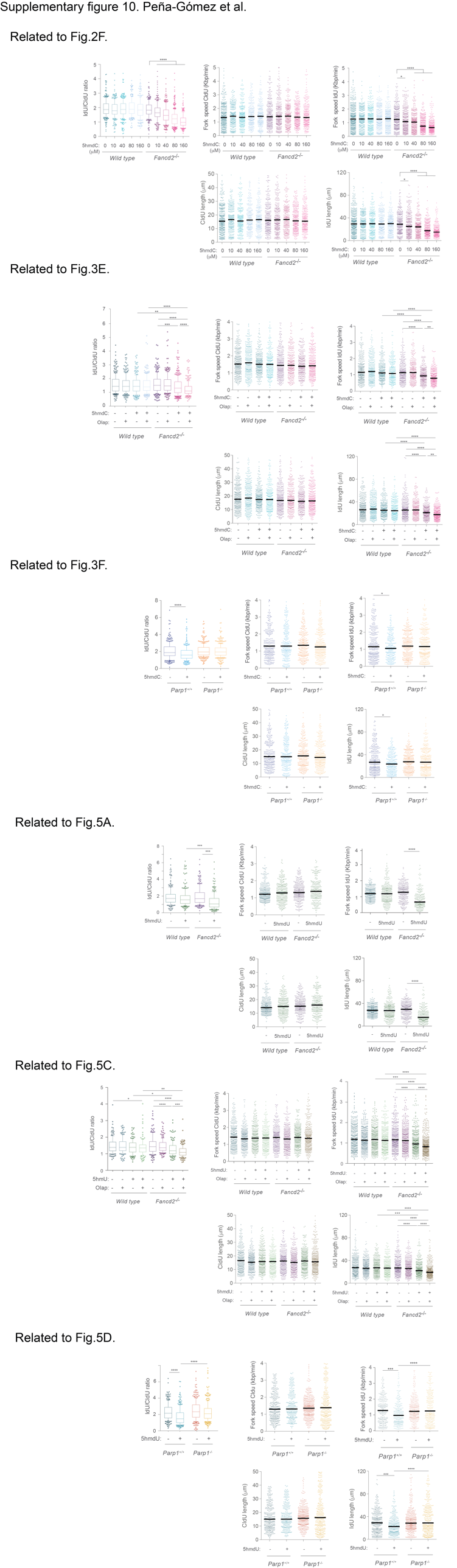

Supplement: Supplementary file 11 — Supplementary figure 10 [file 41419_2022_4952_MOESM11_ESM.png]

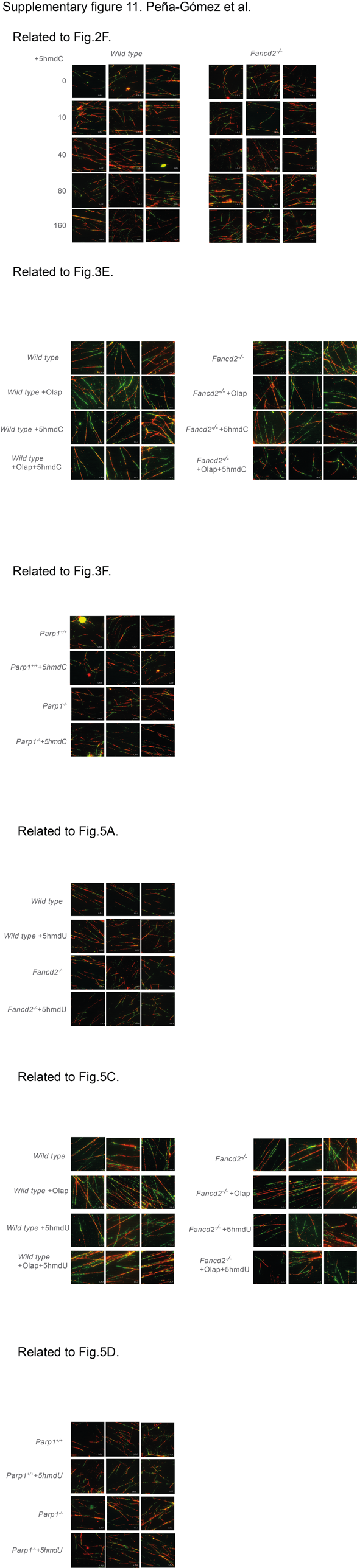

Supplement: Supplementary file 12 — Supplementary figure 11 [file 41419_2022_4952_MOESM12_ESM.png]

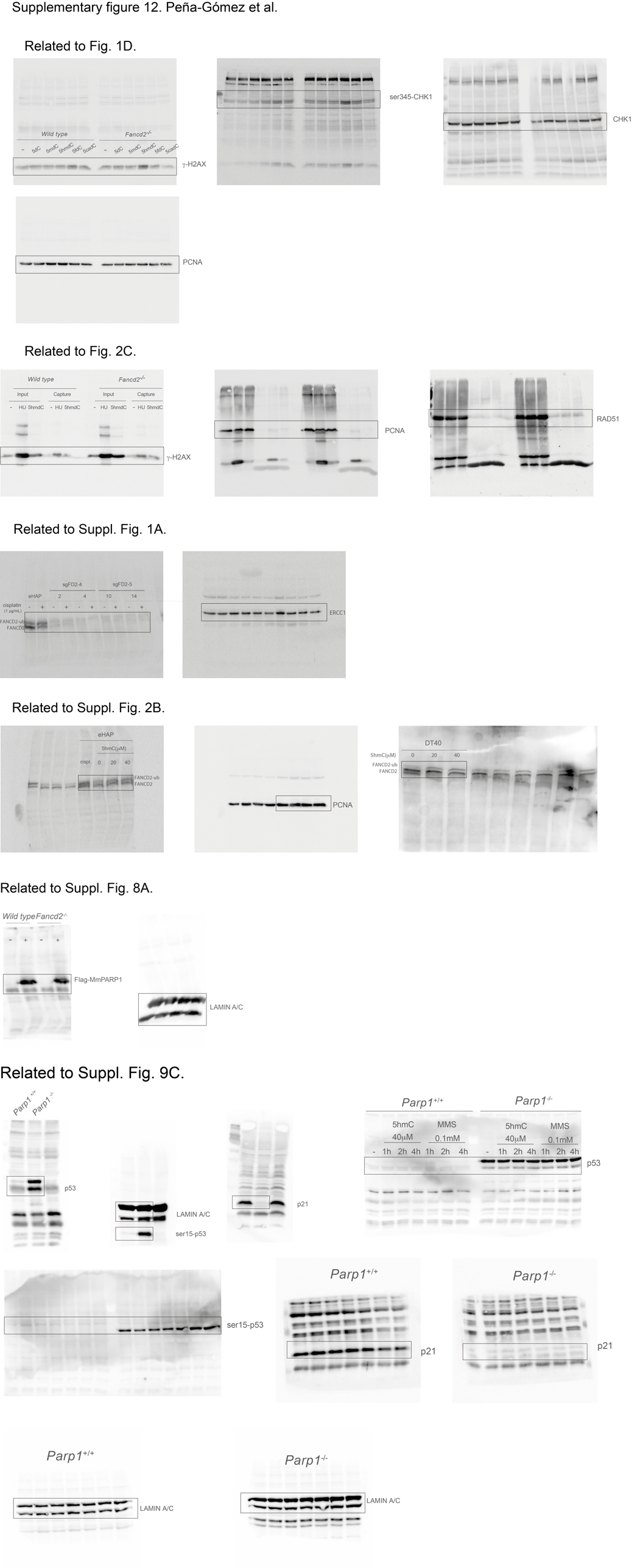

Supplement: Supplementary file 13 — Supplementary figure 12 [file 41419_2022_4952_MOESM13_ESM.png]
